# Individuality and ethnicity eclipse a short-term dietary intervention in shaping microbiomes and viromes

**DOI:** 10.1371/journal.pbio.3001758

**Published:** 2022-08-23

**Authors:** Junhui Li, Robert H. George Markowitz, Andrew W. Brooks, Elizabeth K. Mallott, Brittany A. Leigh, Timothy Olszewski, Hamid Zare, Minoo Bagheri, Holly M. Smith, Katie A. Friese, Ismail Habibi, William M. Lawrence, Charlie L. Rost, Ákos Lédeczi, Angela M. Eeds, Jane F. Ferguson, Heidi J. Silver, Seth R. Bordenstein

**Affiliations:** 1 Vanderbilt Microbiome Innovation Center, Vanderbilt University, Nashville, Tennessee, United States of America; 2 Department of Biological Sciences, Vanderbilt University, Nashville, Tennessee, United States of America; 3 Vanderbilt Genetics Institute, Vanderbilt University Medical Center, Nashville, Tennessee, United States of America; 4 Stanford University Genetics Department, Stanford University, Palo Alto, California, United States of America; 5 Department of Medicine, Vanderbilt University Medical Center, School of Medicine, Nashville, Tennessee, United States of America; 6 Institute for Software Integrated Systems, Vanderbilt University, Nashville, Tennessee, United States of America; 7 Department of Electrical Engineering and Computer Science, Vanderbilt University, Nashville, Tennessee, United States of America; 8 Division of Cardiovascular Medicine, Vanderbilt University Medical Center, Nashville, Tennessee, United States of America; 9 School for Science and Math at Vanderbilt, Collaborative for STEM Education and Outreach, Department of Teaching and Learning, Vanderbilt University, Nashville, Tennessee, United States of America; 10 Vanderbilt Institute for Infection, Immunology and Inflammation, Vanderbilt University Medical Center, Nashville, Tennessee, United States of America; 11 Tennessee Valley Healthcare System, Department of Veterans Affairs, Nashville, Tennessee, United States of America; 12 Department of Pathology, Microbiology, and Immunology, Vanderbilt University Medical Center, School of Medicine, Nashville, Tennessee, United States of America; University of Copenhagen Faculty of Health and Medical Sciences: Kobenhavns Universitet Sundhedsvidenskabelige Fakultet, DENMARK

## Abstract

Many diseases linked with ethnic health disparities associate with changes in microbial communities in the United States, but the causes and persistence of ethnicity-associated microbiome variation are not understood. For instance, microbiome studies that strictly control for diet across ethnically diverse populations are lacking. Here, we performed multiomic profiling over a 9-day period that included a 4-day controlled vegetarian diet intervention in a defined geographic location across 36 healthy Black and White females of similar age, weight, habitual diets, and health status. We demonstrate that individuality and ethnicity account for roughly 70% to 88% and 2% to 10% of taxonomic variation, respectively, eclipsing the effects a short-term diet intervention in shaping gut and oral microbiomes and gut viromes. Persistent variation between ethnicities occurs for microbial and viral taxa and various metagenomic functions, including several gut KEGG orthologs, oral carbohydrate active enzyme categories, cluster of orthologous groups of proteins, and antibiotic-resistant gene categories. In contrast to the gut and oral microbiome data, the urine and plasma metabolites tend to decouple from ethnicity and more strongly associate with diet. These longitudinal, multiomic profiles paired with a dietary intervention illuminate previously unrecognized associations of ethnicity with metagenomic and viromic features across body sites and cohorts within a single geographic location, highlighting the importance of accounting for human microbiome variation in research, health determinants, and eventual therapies.

**Trial Registration:** ClinicalTrials.gov ClinicalTrials.gov Identifier: NCT03314194.

## Abstract 2

Many diseases linked with ethnic health disparities associate with changes in microbial communities in the United States, but the causes and persistence of ethnicity-associated microbiome variation are not understood. This study reveals that individuality and ethnicity eclipse a short-term dietary intervention in shaping microbiomes and viromes.

## Introduction

Composed of trillions of microbial cells and thousands of species, the human microbiome can substantially impact many aspects of human physiology and contribute to chronic diseases underlying health disparities [1–5]. The current drive toward clinical microbiome studies and personalized medicine is, however, hampered by a lack of understanding of the complex social, cultural, and economic causes that contribute to interpersonal differences in microbiome compositions and proportions of specific microbes. Many intrinsic (e.g., age, sex, ancestry) and extrinsic (e.g., lifestyle, diet) factors can associate with microbiome variation, but covariation between factors often confounds their relative importance when studying human populations. For example, investigations statistically disentangle influential factors in large observational studies that are powered to correct for multiple variables at once [4,6–9], but factors such as diet, genetics, geography, and social identities, such as race and ethnicity, often covary in complex, underlying ways that cannot be overcome by increasing sample size alone.

Self-identified race and ethnicity, hereafter referred to as ethnicity, capture aspects of social, cultural, economic, geographic, and historical identity. Ethnicity is not a biological category, but rather a social construct that serves as a proxy for differences in multiple intersecting environmental and social factors and their associated structural drivers, such as racism [10–13]. While observational studies reproducibly link ethnicity with gut [14–18], oral [19,20], and vaginal [21–23] microbiome variation in populations within and between countries, the specific factors underlying these ethnicity associations are not clear. Diet is one of the most widely considered factors shaping gut microbiomes with emerging mainstream appeal [24–28]. While dietary differences can associate with ethnicity, the complexity of factors intertwining ethnicity and dietary regimes indicate observational studies alone cannot confidently disentangle their relative roles in shaping the microbiome, and our previous analysis of dietary metadata in 16S rRNA gene amplicon studies suggested relatively little association between diet and ethnicity-associated microbiome variation in American guts (stool samples) [17]. Notably, microbiome studies that strictly control for diet across ethnically diverse populations are lacking. Thus, we understand little about which extrinsic or intrinsic factors lead to and regulate this variation between ethnicities, and we do not yet understand how the variation within a person over time, or between different people, influences predispositions to wellness and disease.

Ethnicity, encompassing social, environmental, geographic, and cultural variation as well as differential exposure to social and structural discrimination, is a major defining factor of health disparity incidence [29–34]. Diseases associated with microbiomes are also often linked with health disparities across different ethnicities in the United States, such as inflammatory bowel disease, lung cancer, and colorectal cancer [31,33,35,36]. A key question then is whether or not ethnicity-associated microbiome variation at the metagenomic and viromic levels is reproducible across studies and persistent in healthy participants even when diets are the same. Results may in turn lead to consideration of research frameworks and interventions that are more inclusive and actively attentive to ethnic health disparities.

Moreover, multiomic analyses combining metagenomics and metabolomics can provide refined insights into taxonomy, functional potential, and metabolites used or produced by microorganisms that impact health, as in the microbial generation of trimethylamine N-oxide from red meat, which contributes to cardiovascular disease [37–39]. Technological advances and more established reference databases also allow researchers to probe microbiomes in increasingly holistic ways by including metagenomic sequencing of purifed viral particles such as bacteriophages (viruses of bacteria; i.e., phages). Phages often outnumber gut bacteria in this environment, horizontally transfer DNA [40], and restructure microbiomes during lytic events that can occur in response to diet [41–43] and inflammation [40,44].

Controlled studies that limit variation in intrinsic and extrinsic factors are required to associate and disentangle factors such as diet, sex, age, differential exposure to social and environmental determinants of health, and medical history in multiethnic cohorts. At the same time, combining multiomic approaches will help differentiate underlying scales of biology (e.g., microbiome, virome, metabolome) that impact ethnicity-associated variation and health outcomes. The Vanderbilt Microbiome Innovation Center (VMIC) coordinated a human clinical trial to examine whether ethnicity-associated, multiomic variation persists in gut and oral microbiomes (microbial metagenomics), gut viromes (viral metagenomics), and blood and urine metabolites (metabolomics) during a controlled, short-term, dietary intervention. This study details the results.

## Results

### Overview of the clinical trial

The aims of the VMIC study were 2-fold: (i) determine whether ethnicity links with short-term longitudinal, multiomic variation at multiple body sites; and (ii) test directly if ethnicity or diet prevails over the multiomic differences in the microbiome, metabolome, and virome. Thirty-six healthy females who self-reported their ethnicity as Black non-Hispanic or White non-Hispanic (cohort 1: 9 Black/10 White female participants; cohort 2: 7 Black/10 White female participants) enrolled in the study and completed sample collection. Other inclusion criteria controlled for in the study were: age 18 to 40, normal BMI of 18.5 to 24.9 kg/m^2^ at baseline before perturbation, parental ethnicity self-declared to be the same as the participant, not currently pregnant or breastfeeding, no vegetarian or vegan diet, no dietary restrictions, no current tobacco use, and no history of chronic disease or current illness (Trial Registration: ClinicalTrials.gov: NCT03314194; S1 Fig). Stratified participant sampling information is provided in Table C in S1 Table.

Baseline characteristics of the cohorts including age (y), body mass index (BMI) (kg/m^2^), and inflammation marker C-reactive protein (CRP) (mg/dl) did not significantly differ between Black and White individuals in either cohort or within an ethnicity between cohorts (Mann–Whitney U, *P* > 0.05; Table A in S1 Table). Additionally, study participants’ residential 5-digit zip codes were mapped to 2018 county census tracts. County census tracts covered 6 counties in total, although a majority of participants’ zip codes overlapped just 2 adjacent counties. Census tracts were used to retrieve Centers for Disease Control and Social Vulnerability Index (SVI) scores [45] to provide an approximation of representative social exposures, represented as 4 summary themes for socioeconomic, household composition and disability, minority status and language, and housing type and transportation rankings among all tracts in the US. Statistical analysis of these themes did not identify significant differences by ethnicity or individual when controlling for cohort differences (PERMANOVA, r^2^ = 0.3%, *P* = 0.88; Table B in S1 Table). However, we caution that given the homogeneity of census tracts among study participants and participant recruitment occurring primarily on a college campus, SVI themes as applied here may not be representative of individual social and environmental exposures. Further stratified participant sampling information is provided in Table C in S1 Table.

**Fig 1:**
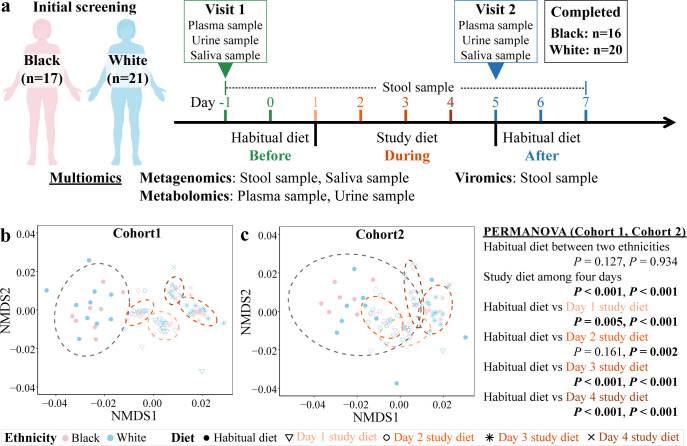
Experimental design and diet profiles. (A) The VMIC study examined short-term longitudinal, multiomic data from multiple body sites over 9 days with 3 broad categories of evaluation (before: habitual diet, days −1 and 0; during: vegetarian diet, days 1 through 4; after: habitual diet, days 5 through 7). (B, C) Nutrient profiles inclusive of macro- and micronutrients (normalized by kcals) of the habitual diet and daily, vegetarian, study diets (Data underlying plot B and C can be found at S1 Data) are shown by NMDS ordination analysis based on Euclidean distance and statistically compared for significant differences by PERMANOVA. Human cliparts in plot A were created with BioRender.com. NMDS, nonmetric multidimensional scaling; PERMANOVA, permutational multivariate analysis of variance; VMIC, Vanderbilt Microbiome Innovation Center.

Habitual nutrient profiles (i.e., macro- and micronutrients) were estimated from self-reported food records using 24-hour dietary recalls (AMPM 5-pass method) via the Nutrition Data System for Research (NDSR) program (http://www.ncc.umn.edu/; 2019), along with detailed diet history and food frequency questionnaires. The nutritional intake was personalized between participants and also profiled in the NDSR program, with calorie needs for participants based on the Mifflin–St. Jeor equation (S2 Data). Nutrient profiles before, during, and after a vegetarian, dietary intervention were normalized by intake calories (kcals per participant’s daily diet). Nearly all daily study diets are significantly different across the 4 days of the vegetarian diet when day’s diet was compared to the participant’s habitual diet (PERMANOVA, *P* < 0.001, Fig 1B and 1C and Table D in S1 Table). Additionally, the nutrient profiles of the habitual diet are not different between the 2 ethnicities (PERMANOVA, cohort 1: r^2^ = 10.5%, *P* = 0.127, cohort 2: r^2^ = 14.3%, *P* = 0.934; Fig 1B and 1C and Table D in S1 Table). Similarly, testing of nutritional profiles between cohorts did not identify differences in the habitual diets between cohorts, although there were cohort differences between the nutrient profiles on day 1 and day 2 of the 2 cohorts in which the nutrient profiles differed (PERMANOVA, *P* < 0.05, Fig 1B and 1C and S1 Data). This source of variation between cohorts could also contribute to some of the differences between cohorts that emerged during the diet.

Parallel analysis of food frequency surveys of participants prior to initiating the study diet did not find significant differences among food intake frequency between ethnicities out of 22 food type groups, except more frequent “sugary sweet food” (*P* = 0.04) intake among Black participants and more frequent water intake >32 oz/day in White participants (*P* = 0.01) [46].

Personalized, multiomic profiles including metabolomics from plasma and urine, microbial metagenomics from gut and saliva, and viromics from gut were collected throughout the study from 36 participants (Fig 1A). Individual DNA was extracted from a total of 225 gut samples and 72 saliva samples collected from the 36 participants who completed the study (Fig 1A and Table D in S1 Table). In cohort 1, an average of 12.7 million (±8.0 million) paired-end reads per gut sample and 4.9 million (±3.1 million) paired-end reads per oral sample were produced for the microbial metagenomes (S3 Data). In cohort 2, an average of 17.0 million (±4.2 million) paired-end reads per gut samples and 15.0 million (±3.8 million) paired-end reads per oral sample were produced for the microbial metagenomes (S3 Data). All gut or saliva microbial metagenomes from the same participant were coassembled into an average of 70,860 gut and 29,478 oral contigs per individual in cohort 1, and 78,823 gut and 36,139 oral contigs per individual. Cesium chloride purified fecal viruses produced an average of 8.9 million (±9.2 million) and 5.6 million (±5.4 million) paired-end reads in cohorts 1 and 2, respectively. Viral metagenomes from each participant across time points were also coassembled and annotated using VirSorter to classify phage sequences above 5,000 bp. In all cases, cohort 1 and cohort 2 were analyzed independently. Subsequently, the results of cohort 2 were used to validate results from cohort 1 such that replication of community differences and individual taxa or genes were considered consistently occurring if identified as significant in both cohorts in the same direction. Additionally, metagenomes were analyzed using an assembly-free approach with HUMAnN (v3.0.0.alpha.4) to confirm taxonomic and functional results and provide comparability to other analyses.

### Individuality dominates microbial and viral community variation

**Fig 2:**
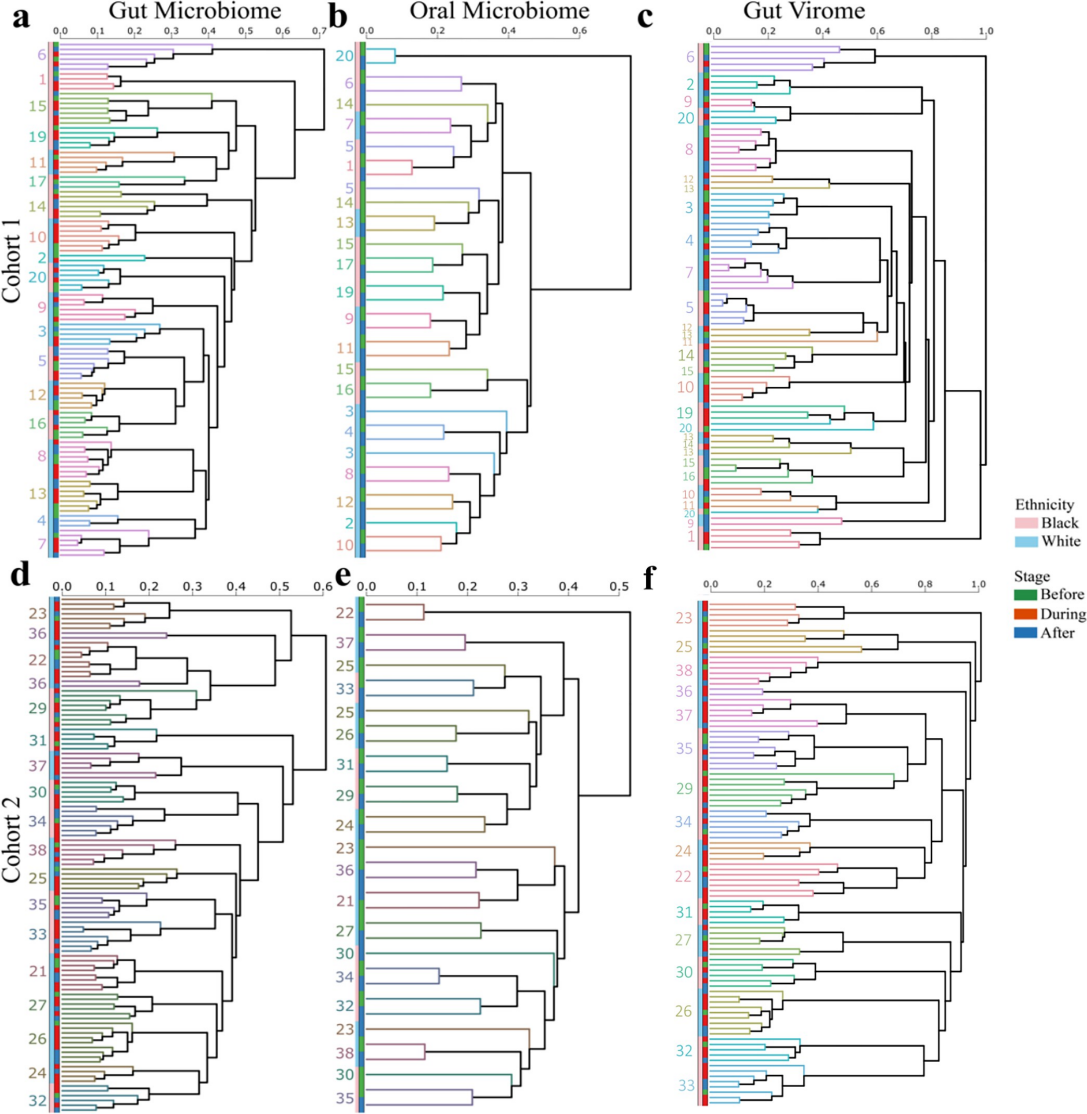
Dendrograms summarizing relationships of microbial community compositions before, during, and after the diet. Participants are individually colored in the branches, and ethnicities (light blue and pink) are denoted at the tips of the branches along with the before (green), during (red), and after (blue) stages of the dietary intervention. Unweighted pair group method with arithmetic mean (UPGMA) clustering trees are shown for the (A, D) gut and (B, E) oral microbial community compositions and (C, F) gut viral community composition in (A-C) cohort 1 and (D-F) cohort 2 based on Bray–Curtis distances (permutations = 999). Data underlying this figure can be found at S1 Data.

We first evaluated the beta diversity relationships and longitudinal clustering of the metagenomic taxonomy profiles in the gut and oral sites using the Bray–Curtis dissimilarity that accounts for presence and abundance of taxa at the strain level. Individual gut microbial community compositions are similar over time as indicated by perfect self-clustering in the gut microbial community dendrogram and the dichotomy of microbiome variation associated with interpersonal versus intrapersonal comparisons between diet intervention stages when both individual identity and dietary intervention stage were both included in the model (cohort 1: r^2^ = 86.6% *P* < 0.001 versus r^2^ = 0.6% *P* < 0.001; cohort 2: r^2^ = 88.3% *P* < 0.001 versus r^2^ = 0.5% *P* = 0.07, PERMANOVA with marginal sums of squares, Fig 2A and 2D and Table A in S2 Table). Similarly, in the oral microbial community composition, self-clustering in the dendrogram is persistent with 2 exceptions, and interpersonal comparisons again account for more variation than intrapersonal comparisons (cohort 1: r^2^ = 86.9% *P* < 0.001 versus r^2^ = 0.8% *P* = 0.296; cohort 2: r^2^ = 85.2% *P* < 0.001 versus r^2^ = 1.3% *P* = 0.127, PERMANOVA with marginal sums of squares, Fig 2B and 2E and Table A in S2 Table). Similar results were found with the assembly-free analysis (Table B in S2 Table). Gut virome analyses exhibit the same trend in the dendrogram, but with less self-clustering in cohort 1 compared to cohort 2 (r^2^ = 71.0% versus r^2^ = 88.8%, Fig 2C and 2F and Table A in S2 Table), and interpersonal comparisons again associate with more of the variation than intrapersonal comparisons (Fig 2C and 2F and Table A in S2 Table). Similar results for all 3 communities in both cohorts were observed with the Binary Jaccard distance that takes into account only presence of taxa or viral contigs (S1 Data). Due to the strong interindividual variation and including multiple samples from each individual in the analysis, all statistical models used sampling days as strata to account for both individuality and the day/stage of the diet intervention.

### Ethnicity-associated variation in metagenomic communities and functional potential persists during and after shared diets

#### Taxonomic diversity

**Fig 3:**
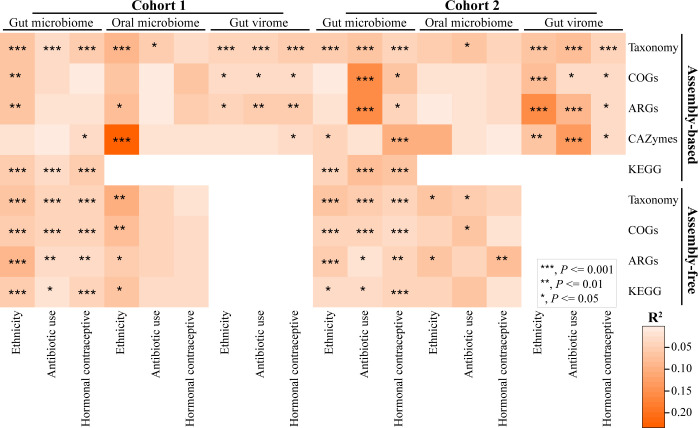
Ethnicity-associated variation in metagenomic communities and functions. The gradient of colors denotes variation explained by the variables, and asterisk symbol indicates *p*-value of PERMANOVA with Bray–Curtis distance that was performed at all combined dietary stages for taxonomy at the strain level, COGs, ARGs, and CAZymes [peptidoglycanase for virome]. adonis2(data ~ Ethnicity + Antibiotic Use + Hormonal Contraceptive, permutations = perm, method = “bray”, by = “margin”), where perm = with (data, how(nperm = 999, blocks = Day)). See S2 Fig for details of functional categories used for assembly-based PERMANOVA. Data underlying this figure can be found at S1 Data. ARG, antibiotic-resistant gene; CAZymes, carbohydrate active enzymes; COG, cluster of orthologous groups; PERMANOVA, permutational multivariate analysis of variance.

Despite similar macro- and micronutrient intake during the vegetarian diet, gut and oral microbial community compositions consistently varied between ethnicities across both cohorts and persisted throughout the shared diet stage and return to habitual diets. Variation was detected in strain-level taxonomic composition using Bray–Curtis dissimilarities (cohort 1 gut: r^2^ = 6.2%, *P* < 0.001, PERMANOVA with marginal sums of squares, Fig 3; cohort 1 saliva: r^2^ = 9.1%, *P* < 0.001, PERMANOVA with marginal sums of squares, Fig 3; cohort 2 gut: r^2^ = 5.6%, *P* < 0.001, PERMANOVA with marginal sums of squares, Fig 3; cohort 2 saliva: r^2^ = 5.4%, *P* < 0.001, PERMANOVA with marginal sums of squares, Fig 3 and S1 Data) and Binary Jaccard distances (S1 Data) when controlling both for individuality and dietary intervention stage. A test for homogeneity of variance between ethnicities was only significant for gut samples in cohort 1 with Bray–Curtis metrics from both assembly-based and assembly-free analyses (S4 Table), indicating that between-group differences in dispersion may be contributing to the significant differences in community composition in gut microbiome samples in this cohort. Additionally, the dietary intervention caused a subtle statistically significant shift in the gut microbial community composition. This effect of diet was observed inconsistently in viral community composition between cohorts, and not in any oral microbial community compositions (S2 Table), a result largely consistent with some prior studies discussed below. The Shannon index alpha diversities for microbial species richness and evenness were generally similar across sites in both cohorts with few exceptions (S5 Table). In addition to the stable ethnicity-associated variation pattern in the microbial communities, there is significant variation in ethnicity-associated beta diversity in the viral communities throughout the study in cohort 1 (r^2^ = 4.5%, *P* < 0.001, PERMANOVA with marginal sums of squares, Fig 3 and S1 Data) and cohort 2 (r^2^ = 6.1%, *P* < 0.001, PERMANOVA with marginal sums of squares, Fig 3 and S1 Data) when controlling for individuality and dietary intervention stage. Variances were mixed between ethnicities in all viral communities with heterogeneity detected in cohort 1 (cohort 1: Bray–Curtis: *P* = 0.020; cohort 2: Bray–Curtis: *P* = 0.140, Tables A and B in S4 Table).

To determine if the dietary intervention caused a gradual lessening of microbiome dissimilarity between ethnicities, we compared intragroup and intergroup oral and gut microbiome compositions through permutational pairwise testing of beta dispersions with Bray–Curtis dissimilarity in the assembly-free data. We performed this analysis for both inter- and intragroup distances by study day and stage, although some samples within an ethnicity and day were small and could not be computed. In this analysis, there were no significant differences between days or stages in either oral or gut microbiomes (Permutation test of multivariate homogeneity of groups dispersions, *P* > 0.05, Tables J and K in S2 Table and S4 Data), which suggests that groups began with different microbiome compositions and remained different over each day of the study without a detectable convergence as the study progressed. Taken together with results from the Fig 2 dendrograms, we conclude that individual and ethnicity-associated variation in microbial and viral community taxonomic compositions are generally persistent and recurrent across datasets before, during, and after the diet. We discuss specific taxon differences below.

#### Function

We next compared 4 aspects of functional potential of the gut and oral microbial metagenomes and gut viral metagenomes: clusters of orthologous groups (COGs), KEGG orthologs (KOs), carbohydrate active enzymes (CAZymes), and antibiotic resistant genes (ARGs). First, assembly-based analyses of the composition of functional potential based on gut, oral, and viral metagenomic COGs identify significant degrees of ethnicity-associated variation based on COG compositional data when accounting for day and repeat sampling in gut microbiomes in cohort 1 (r^2^ = 7.1%, *P* = 0.003; PERMANOVA with marginal sums of squares, Fig 3 and S1 Data) and gut viromes in both cohorts (r^2^ = 7.1% and 8.4%, *P* < 0.001, PERMANOVA with marginal sums of squares, Fig 3 and S1 Data). Tests for homogeneity of variance between ethnicities were significant for gut samples in both cohorts (Tables A and B in S4 Table), indicating that between-group differences in dispersion may be contributing to the significant differences in COG composition in the gut microbiome. Similar associations of ethnicity with COG compositional data were found in the assembly-free analysis with ethnicity accounting for 4.4% to 8.0% of the variation (Fig 3 and S1 Data).

Second, there were different KO compositions between the ethnicities in the gut microbiome from all combined stages using contig data (cohort 1: r^2^ = 5.3%, *P* < 0.001; cohort 2: r^2^ = 3.8%, *P* < 0.001; PERMANOVA with marginal sums of squares, S1 Data and Fig 3) and the assembly-free approach (cohort 1: r^2^ = 5.8%, *P* < 0.001; cohort 2: r^2^ = 3.4%, *P* = 0.013; PERMANOVA with marginal sums of squares, Fig 3 and S1 Data). In contrast, dietary stage was weakly and inconsistently associated with KO compositional variation in the gut microbiome in both cohorts (Tables H and I in S2 Table). Variances were consistently homogeneous across diet stages from KO compositions in each cohort based on both assembly-based and assembly-free approaches (S3 Table). Tests for homogeneity of variance between ethnicities were significant for gut samples in cohort 1 but not in cohort 2, irrespective of assembly-based and assembly-free approaches, while variances were consistently homogeneous across ethnicities for oral KO compositions in each cohort based on assembly-free approach data (S4 Table), indicating that between-group differences in dispersion may be contributing to the significant differences in gut KO compositions in cohort 1.

We explored the composition of KOs, which were significantly enriched between ethnicities within each stage of the diet and in both cohorts through the univariate statistical analysis and manual annotation of KOs (S5 Data). Prior to the diet, 3 significantly enriched KOs appeared in both cohorts, 2 of which were consistently more enriched in Black individuals and mapped to heat shock protein and tRNA pathways. Within the controlled dietary intervention stage, 113 KOs overlapped in the same direction and ethnicity across cohorts. Among those 113 KOs, 53 mapped to enzymatic pathways most of which related to microbial metabolism with repeated observation of functions including oxidoreductases, transferases, hydrolases, and lyases, while another 23 mapped to transporter pathways with 12 representing ABC transport systems and 5 representing ion channel pores. Examination of the enzyme functions reveals many share similar roles in nucleotide, nicotinate and nicotinamide, and NAD+ oxidative phosphorylation metabolism. Two CRISPR-associated pathway proteins were enriched in White individuals in both cohorts, and while the KO is annotated as a prokaryotic defense system, it was discovered in extremophilic archaea. Additional pathway functions that appeared but were less frequently enhanced include transcription and translation factors, translation and tRNA modification factors, DNA repair and recombination proteins, and bacterial motility proteins. The nicotinate and nicotinamide metabolism pathways interrelate with cofactor and B-vitamin metabolism and specifically NAD biosynthesis, a major redox and signaling molecule during oxidative phosphorylation. In total, 84 of the 113 KOs were enriched in Black individuals with 29 enriched in White individuals, and 17 were poorly characterized at the pathway level. The post-dietary stage had 15 enriched KOs in the same direction for ethnicity and cohort, with 13 enriched in Black individuals and 2 enriched in White individuals. The same transporter and enzyme categories were again represented, but a greater diversity of functions were also enriched including heat shock and ribosomal proteins and tRNA modification factors. Overall enrichment differences in KOs primarily represent categories related to microbial metabolism and energy production, and significantly more enriched pathways were observed during (*N =* 113) the dietary stage than before (*N* = 2) or after (*N* = 15).

Third, CAZymes annotated from CAZyme categories (S6 Data) involved in the degradation of carbohydrates were analyzed compositionally across combined stages for each body site. There is significant, ethnicity-associated variation in the total composition of CAZymes in the gut and saliva in both cohorts (Fig 3), while the dietary intervention once again did not influence CAZyme compositions (Table G in S2 Table). Tests for homogeneity of variance between ethnicities was significant for both gut and oral samples in cohort 1 but not in cohort 2 (Tables A and B in S4 Table), indicating that between-group differences in dispersion may be contributing to the significant differences in gut and oral CAZyme compositions in cohort 1. Ethnicity accounts for 23.4% of the total oral CAZyme compositional variation in cohort 1 (*P* < 0.001, PERMANOVA with marginal sums of squares, Fig 3 and S1 Data) and nearly 10% of the total oral CAZyme composition variation in cohort 2 (*P* = 0.055, PERMANOVA with marginal sums of squares, Fig 3 and S1 Data) when controlling for individuality and dietary intervention stage. Analysis of total compositional variation can mask finer resolution differences in the CAZyme categories. Focusing on abundance differences of each of the 25 CAZyme categories identified in cohort 1, 8 gut and 18 oral categories were significantly different between ethnicities across all combined stages (*P*_FDR_ < 0.05, Wilcoxon rank-sum test, Figs A and B in S2 Fig). In cohort 2, 1 gut and 21 oral CAZyme categories were significantly different between ethnicities across all combined stages (*P*_FDR_ < 0.05, Wilcoxon rank-sum test, Figs C and D in S2 Fig). Of the 21 oral CAZymes categories that were differentially abundant in cohort 2, 15 were also significantly different between ethnicities in cohort 1. Of these 15 oral CAZyme categories, CAZymes associated with fiber substrates (e.g., cBeta-glucans, Cellulose, cCellulose, Lignin, Xylan, and cXyloglucan) tended to be more abundant in White individuals, while CAZymes associated with sugar substrates (e.g., Dextran, Fructan, cMannan, and Mannan) were more abundant in Black participants (Figs B and D in S2 Fig). Analysis of the gut virome peptidoglycanase that is involved in the degradation of peptidoglycan (i.e., the major components of bacterial cell wall) during phage lysis also indicated ethnicity-associated variation in both cohorts (Fig 3 and Figs E and F in S2 Fig).

Fourth, we analyzed differences in ARGs using hmmscan [47] by assigning reads to Resfams functional categories of existing and putative ARGs [48]. Across all combined stages, there was significant ethnicity-associated variation in gut and oral microbial ARGs and in gut viral ARGs in cohort 1, accounting for 5.9% to 8.1% of the total ARG compositional variation (*P* < 0.01, PERMANOVA with marginal sums of squares, Bray–Curtis distance, Fig 3 and S1 Data), but there was no significant ethnicity-associated variation in gut and oral microbial ARGs in cohort 2 (*P* > 0.05, PERMANOVA with marginal sums of squares, Fig 3 and S1 Data). Nevertheless, ethnicity explained 13.8% of the total ARG compositional variation in the gut virome in cohort 2 (*P* < 0.001, PERMANOVA with marginal sums of squares, Fig 3 and S1 Data). Tests for homogeneity of variance between ethnicities were significant for gut samples in cohort 1 and oral samples in cohort 2 (Tables A and B in S4 Table), indicating that between-group differences in dispersion may be contributing to the observed differences in ARG compositional variation. Dietary stage was not associated with ARG compositional variation in the gut microbiome in cohort 2, as well as with ARG compositional variation in the oral microbiome in cohort 1 (*P* > 0.05, PERMANOVA with marginal sums of squares, Tables E and F in S2 Table). ARG compositions were not associated with dietary stage in either gut virome cohort (cohort 1: r^2^ = 0.6%, *P* = 0.91; cohort 2: r^2^ = 0.8%, *P* = 0.31; PERMANOVA with marginal sums of squares, Table E in S2 Table). The assembly-free analysis of gut samples supported the findings of cohort 1, with ethnicity accounting for 5.4% to 8.5% of the variation in ARG composition, whereas ethnicity also had significant associations with both gut and oral microbial ARG compositional variation in the assembly-free approach in cohort 2 (Fig 3).

#### Assembly-free comparisons between cohorts

In order to examine the effect of cohort, we performed a combined analysis of both oral and gut bacterial microbiome cohorts using the assembly-free approach. Cohort was significantly associated with gut and oral taxonomic compositions, gut and oral ARG compositions, and gut COG composition (S3 Table). Variances were heterogeneous between cohorts for COG composition only (Table E in S4 Table). The strength of the effect of cohort on taxonomic composition was similar to that of ethnicity (gut: 3.7% *P* < 0.001 versus 3.3% *P* < 0.001; saliva: 2.3% *P* < 0.001 versus 5.5% *P* = 0.048; PERMANOVA with marginal sums of squares, Bray–Curtis distance, S3 Table), as was the strength of the effect of cohort on ARG composition (gut: 4.2% *P* < 0.001 versus 2.2% *P* < 0.001; saliva: 3.4% *P* = 0.006 versus 3.1% *P* = 0.021; PERMANOVA with marginal sums of squares, Bray–Curtis distance, S3 Table). The effect of cohort was much stronger than ethnicity for gut COG composition (4.8% *P* < 0.001 versus 1.9% *P* < 0.001; PERMANOVA with marginal sums of squares, Bray–Curtis distance, S3 Table), whereas cohort had no significant effect on oral COG composition (2.8% *P* = 0.061 versus 3.5% *P* = 0.019; PERMANOVA with marginal sums of squares, Bray–Curtis distance, S3 Table).

### Ethnicity associations are common and persistent for abundant microbial taxa and phages

**Fig 4:**
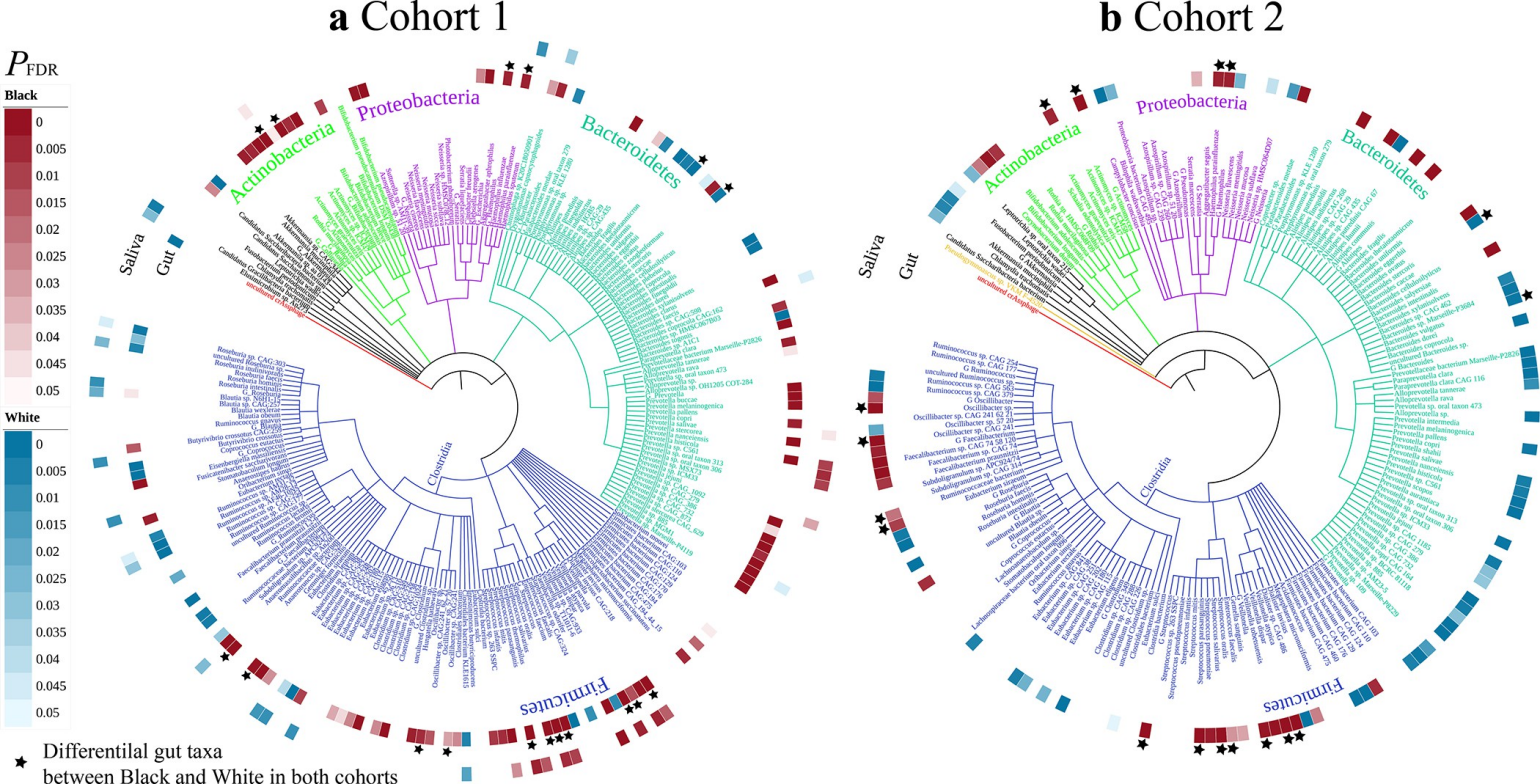
Ethnicity-associated variation in abundant microbial taxa and phage. The 219 taxa in cohort 1 (A) and 182 taxa in cohort 2 (B) included in the phylogeny are present in all participants and have relative abundances of >1% in at least one of the gut or oral microbial metagenomes. The inner circle indicates differential taxa in the gut between the 2 ethnicities; the outer circle indicates differential taxa in the saliva between the 2 ethnicities. Pink or blue color gradients indicate FDR-adjusted *p*-value of significance (LinDA function in MicrobiomeStat package fitting linear mixed-effects models ‘~ Ethnicity + Antibiotic Use + Hormonal Contraceptive + (1|Day)’) for centered log-ratio transformed abundance between the 2 ethnicities. Data underlying this figure can be found at S1 Data. Pink indicates more abundant in Black participants, and blue indicates more abundant in White participants. Star indicates differential abundant taxa in the gut between the 2 ethnicities in both cohorts. Taxon name with a “G” before the name indicates the taxon was classified at the genus level.

A total of 219 abundant taxa in cohort 1 and 182 abundant taxa in cohort 2 at or above the genus level are detected at >1% relative abundance in at least one of the gut or oral microbial metagenomes. The phylogenetic relationships of all abundant taxa are shown in Fig 4. Overall, the abundant taxa in these 2 body sites are compositionally different, as expected. Within each of the sites, the relative abundance of highly abundant taxa correlates positively with the relative abundance of that same taxa in the other site for both saliva (r = 0.63) and gut taxa (0.58, *P* < 0.0001, Spearman, S3 Fig), respectively. Among the 219 abundant taxa in cohort 1, a striking 49.3% (108/219) in the gut and 15.5% (34/219) in the oral microbial metagenomes vary in abundance between ethnicities (*P*_FDR_ < 0.05, LinDA, Fig 4A). Similarly, in cohort 2, 49.5% (90/182) of taxa in the gut vary in abundance between ethnicities (*P*_FDR_ < 0.05, LinDA, Fig 4B and S1 Data); oral taxa from cohort 2 are not differentially abundant in Black or White individuals at the same cutoff (*P*_FDR_ < 0.05, LinDA, Fig 4B). While a similar number of taxa were differentially abundant between ethnicities in cohort 1 and cohort 2, only 17 taxa both replicated across cohorts and were differentially abundant in the same direction (*Actinomyces oris*, G_*Actinomyces*, *Bacteroides stercoris*, *Bacteroides vulgatus*, *Eubacterium siraeum*, *Haemophilus parainfluenzae*, *Oscillibacter* sp., *Oscillibacter* sp. CAG_241, Ruminococcaceae bacterium, *Streptococcus oralis*, *Streptococcus parasanguinis*, *Streptococcus salivarius*, G_*Streptococcus*, *Veillonella atypica*, *Veillonella dispar*, G_*Veillonella*, and G_*Haemophilus*). Of these 17 replicating taxa, 15 were more abundant in Black individuals, and 2 *Bacteroides* taxa were more abundant in White individuals (Fig 4). An additional 14 taxa were identified as differentially abundant in both cohorts, but the directionality of the difference was reversed in cohort 2 compared with cohort 1. Smaller proportions of species were differentially abundant between ethnicities in the assembly-free analysis for fecal samples (cohort 1: 29.7% (97/338); cohort 2: 10.6% (31/292); S7 Data). We previously showed that gut taxa also varied by ethnicity in analyses of the American Gut Project (16.2%) and the Human Microbiome Project (20.6%) [17].

In addition to bacteria, crAss-like bacteriophages are the most abundant phages in the gut [49], and in the gut virome in cohort 1, crAssphage, which infects *Bacteroides*, was the most abundant viral species across both ethnicities and significantly higher in White individuals by 1.2-fold. In cohort 2, *Faecalibacterium* phages were collectively the most abundant phage species in all individuals but without significant abundance differences between ethnicities. Overall, 1,272 out of 4,423 (28.8%) total viral contigs are predicted to infect 85 bacterial genera in cohort 1. In cohort 2, 845 out of 2,971 (28.4%) were predicted to infect 64 bacterial genera.

In cohort 1, 11 significantly different viral genera were detected between the 2 ethnicities when accounting for the day of the study (*P*_FDR_ < 0.05, LinDA, S8 Data), 8 were more abundant in White participants with 1 unique phage predicted to infect *Paraprevotella*. In addition, 3 were more abundant in Black participants with 2 phages predicted to infect *Anaerobutyricum* and *Coprobacter* that are specific to Black participants. The largest fold-change observed in the either cohort was for phages of the *Veillonella* genus, which was 1,281-fold more abundant among Black individuals in cohort 1 and specific to Black individuals in cohort 2.

In cohort 2, 6 differentially abundant genera were identified between ethnicities when accounting for the day of study (*P*_FDR_ < 0.05, LinDA, S8 Data). *Coprobacter* was the only consistent finding between cohorts, being specific to Black individuals in cohort 1 and 6.4-fold more abundant in cohort 2. A single phage predicted to infect *Anaerotruncus* was specific to Black participants, while 2 phages predicted to infect *Phascolarctobacterium* and *Blastocystis* were specific to White individuals. The largest difference among phages was 13.4-fold for *Parabacteroides* phages in Black individuals in cohort 2.

### Antibiotic history and hormonal contraceptive use are associated with microbial taxonomic and function composition

Though medication use within the past 3 months was an exclusion criterion, the use of antibiotics within the past year and hormonal contraceptives at the time of sampling were independently associated with microbiome variation (Fig 3 and S1 Data). Based on Bray–Curtis distance of assembly-based analysis, the use of antibiotics within the past year accounted for 1.5% to 7.9% of the total variance in the gut and oral microbial community composition (*P* < 0.05, PERMANOVA with marginal sums of squares, Fig 3 and S1 Data); COGs and ARGs were associated with antibiotic use in gut samples in cohort 2, but not in oral samples in both cohorts (PERMANOVA with marginal sums of squares, Fig 3 and S1 Data); KEGG composition was associated with antibiotic use in gut samples in both cohorts (cohort 1: r^2^ = 3.4%, *P* < 0.001; cohort 2: r^2^ = 8.3%, *P* < 0.001; PERMANOVA with marginal sums of squares, Fig 3 and S1 Data), whereas CAZymes were not associated with antibiotic use in both oral and gut samples in either cohorts (PERMANOVA with marginal sums of squares, Fig 3 and S1 Data), suggesting prior antibiotic usage impacts both taxonomy and specific functional categories of the metagenome. As antibiotic usage may co-correlate with ethnicity, we tested for differences in antibiotic use in cohort 1 and found a marginal nonsignificant difference between groups (*P =* 0.092, χ^2^ = 2.8, χ^2^ test). Antibiotic usage in cohort 2 was not significantly different between ethnicities (*P =* 0.969, χ^2^ = 0.249, χ^2^ test). The use of hormonal contraceptive was also associated with variation in the gut but not oral microbial community composition in cohort 1 (cohort 1: r^2^ = 5.1%, *P* < 0.001; cohort 2: r^2^ = 4.7%, *P* < 0.001; PERMANOVA with marginal sums of squares, Fig 3 and S1 Data). Likewise, the composition of functions were significantly associated with hormonal contraceptive use only in gut samples, i.e., COGs (cohort 2: r^2^ = 6.0%, *P* = 0.014, PERMANOVA with marginal sums of squares, Fig 3 and S1 Data), ARGs (cohort 2: r^2^ = 4.2%, *P* = 0.017, PERMANOVA with marginal sums of squares; Fig 3 and S1 Data, CAZymes (cohort 1: r^2^ = 3.2%, *P* = 0.044; cohort 2: r^2^ = 10.0%, *P* < 0.001; PERMANOVA with marginal sums of squares, Fig 3 and S1 Data) and KEGG (cohort 1: r^2^ = 5.9%; cohort 2: r^2^ = 6.5%; *P <* 0.001, PERMANOVA with marginal sums of squares, Fig 3 and S1 Data). There was no significant association of hormonal contraception with ethnicity in either cohort (cohort 1: *P =* 0.67, χ^2^ = 0.18, χ^2^ test; cohort 2: *P =* 1, χ^2^ = 0, χ^2^ test). The assembly-free analysis from both cohorts supported the findings of antibiotic use and hormonal contraceptive use on the microbial community compositions.

### Ethnicity associations occur for heritable taxa

Our previous analysis uncovered several gut taxa that consistently and significantly varied in abundance between ethnicities based on 16S rRNA gene amplicon sequencing from the American Gut and Human Microbiome Project [17]. Most of these recurrently varying taxa were reported to be heritable and associated with human genetic variation, which tentatively suggested human genotype may contribute to ethnicity-associated variation. Here, we identified 9 of the same bacterial taxa in the metagenomic datasets from both cohorts and tested if they also differ between ethnicities. Pairwise abundance tests validate 2 out of 9 oral taxa in cohort 1 are differentially abundant (*Veillonella* genus and Victivallaceae family, *P*_FDR_ < 0.08, LinDA, S6 Table), and 2 out of 9 gut taxa in cohort 2 are differentially abundant (Christensenellaceae and Rikenellaceae families, *P*_FDR_ < 0.05, LinDA, S6 Table). The 2 families identified in cohort 2 have higher abundances in Black participants, including the Christensenellaceae family that is the most highly heritable taxon in the gut microbiome, and higher abundances are positively associated with several health traits including obesity and inflammatory bowel disease [50–52]. The higher abundances of Rikenellaceae and Christensenellaceae families are associated with reduced visceral adipose tissue and healthier metabolic profile [53].

Pairwise abundance tests also reveal, for the first time, that 2 of the 9 heritable taxa previously identified in the gut are also differentially abundant in the oral microbiome in cohort 1 (*P*_FDR_ < 0.08, LinDA, S6 Table), though they are not present in the gut of cohort 1 participants. One of these taxa, the *Veillonella*, is positively associated with dental calculus [54] and smoking [55]. The *Veillonella* genus is lower in Black participants in cohort 1, which is associated with a notably increased abundance of purified phages predicted to infect the genus *Veillonella* in both cohorts (*P*_FDR_ *<* 0.05, LinDA). Thus, lytic activity of the phages may drive the reduced abundance of *Veillonella*. Phages predicted to infect 3 *Veillonella* spp. (*Veillonella parvula*, *Veillonella* sp. 3110, and *Veillonella* sp. AF36-20BH) are unique in Black participants. The highly heritable gut taxa Christensenellaceae is not differentially abundant in the saliva. In cohort 2, no heritable taxa were identified as differentially abundant in the oral microbiome (S6 Table).

### Metabolomes reflect dietary changes in both ethnicities

**Fig 5:**
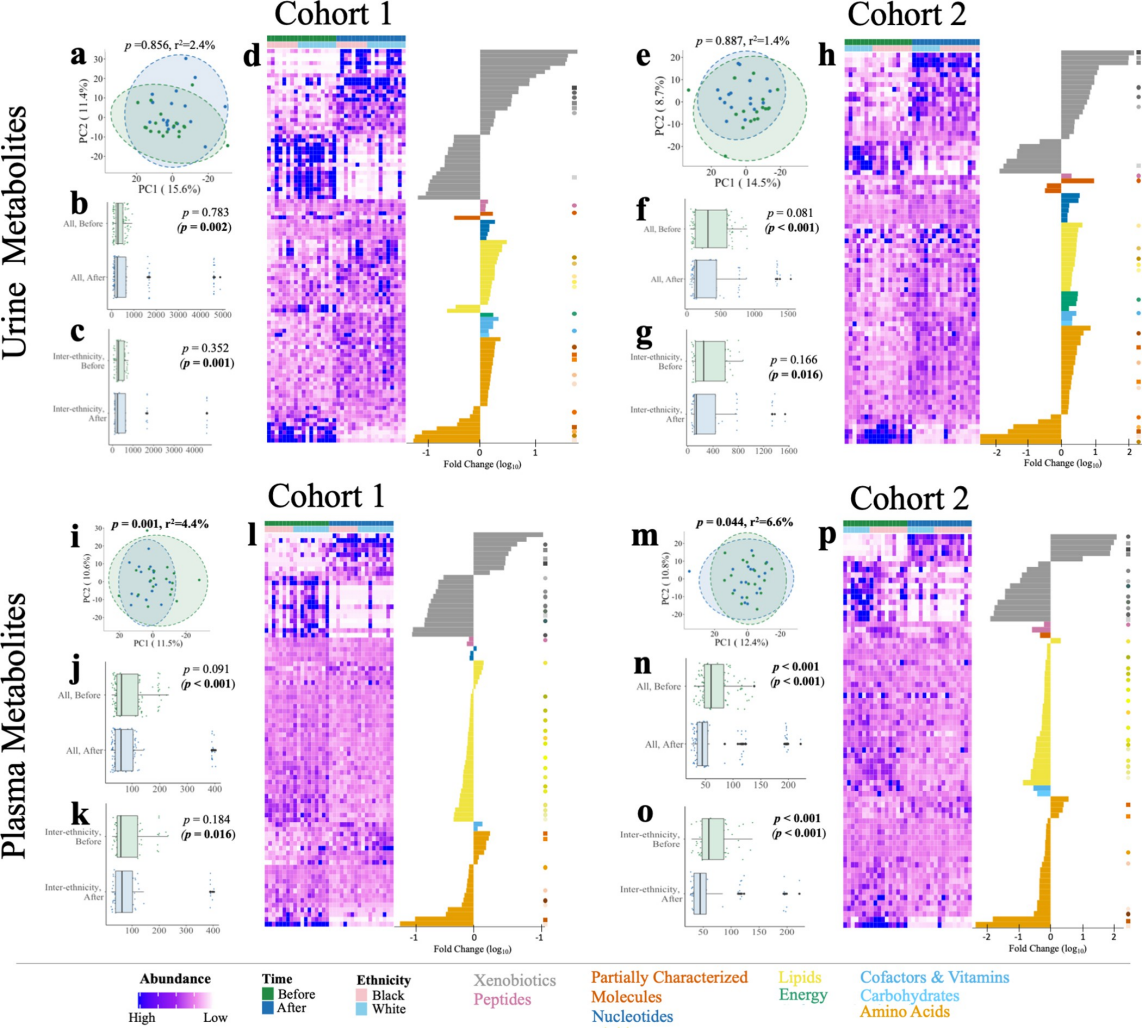
Dietary intervention alters urine and plasma metabolomes. Univariate analysis on metabolomic samples before and after a short-term diet identified 180 significantly changed total metabolites in cohort 1 and 152 metabolites in cohort 2 (*P*_FDR_ < 0.05, Wilcoxon signed-rank test). Total urine metabolome compositions were not significantly different between diet stages or ethnicities in either cohort (A, E) (*P*_FDR_ < 0.05, PERMANOVA). In cohort 1, 97/842 urine metabolites (D) significantly changed over the diet period, while in cohort 2, 80/835 urine metabolites (H) significantly changed. Total plasma metabolome compositions were significantly different between diet phases (I, M) in both years (*P*_FDR_ < 0.05, PERMANOVA). In cohort 1, 83/796 plasma metabolites (L) significantly changed between diet phases, while in cohort 2, 72/736 plasma metabolites (P) were significantly different. Pairwise Euclidean distance between all participants and interethnic groups significantly changed in urine (B, C, F, G) and plasma (J, K, N, O) across cohorts (*P* < 0.05, Wilcoxon rank-sum test). Statistics in parentheses denote the removal of outlier participants (see Materials and methods, Metabolomics analysis*)*. Individual scaled abundances are shown above by stage and by ethnicity and ordered by super pathway. Heatmaps reflect all significantly changed metabolites within cohort-matched, color-coded dots and ordered by decreasing log 10-fold change within each super pathway. Circles denote metabolites significant in both cohorts; squares represent metabolites significantly different in both cohorts and in both urine and plasma. Abundance corresponds to scaled and transformed intensity value of each metabolite. A key of ordered metabolites, fold changes, and *p*-values can be found in S1 Data.

Metabolomic profiles of plasma and urine metabolites were sampled before and after the dietary intervention to proxy the relative importance of ethnicity versus diet. We tested the measured subset of metabolites from before and after the diet between ethnicities to determine if they ran parallel to observed differences in the gut microbiomes. However, the measured subset of metabolites does not appear to differ between ethnicities either before or after the diet (Fig 5). Due to the limitations of the “nontargeted” panel of metabolites, which represent only a subset of all plasma and urine metabolites and not the global metabolome, we cautiously interpret that changes are not observed between ethnicities for the observed metabolomic profiles. However, the reported metabolites were only the most abundant metabolites from a diverse totality of known and unknown compounds.

Given the limitations of quantifying the global metabolome with this approach, we further investigated the magnitudes of both the effect size and statistical difference between metabolites between ethnicities within each dietary stage and cohort. While a number of metabolites demonstrate change between ethnicities at each stage of the diet, no metabolites at the individual level or at the super pathway class (i.e., peptides, lipids, carbohydrates, and amino acids) were statistically different between ethnicities in either urine or plasma in either cohort (S4 and S5 Figs and S1 Data and S7 Table).

The most consistent result, and in general contrast to the microbial and viral metagenomes, is that dietary stage associates with variation in the composition of the detected plasma metabolites (Figs 5I-5K, 5M-5O and S4), and in detected urine metabolites if outliers are removed (Figs 5A-5C, 5E-5G, and S4 and S1 and S9 Data files). This effect is observed independently in each cohort of the study. To identify individual metabolites changed by the dietary intervention, univariate analysis on samples before and after intervention identified a total of 180 significantly changed metabolites spanning 11.5% (97/842) and 10.4% (83/796) of the urine and plasma metabolomes in cohort 1 (*P*_FDR_ < 0.05, Wilcoxon signed-rank test, Fig 5 and S1 Data). Similarly, 152 metabolites significantly changed in cohort 2 of this study, including 9.6% (80/835) of urine metabolites and 9.8% (72/736) of plasma metabolites (*P*_FDR_ < 0.05, Wilcoxon signed-rank test, Fig 5 and S1 Data). Again, we caution that these metabolites represent the most abundant and prevalent entities of the metabolome and therefore preclude a global interpretation of the effect of the dietary change on the metabolome. Given this, within the urine and plasma metabolomes, 26 and 37 of the significantly different metabolites occur in both cohorts, respectively. Among these cross-cohort measured metabolites, 8 significantly changed in both urine and plasma. These 8 metabolites reflect dietary restrictions including the caffeine metabolism pathway (methylxanthine) and the methylhistidine metabolism pathway (3-methylhistidine) related to reduced animal protein input, respectively, and the inclusion and metabolism of allium vegetables, including garlic and onions (alliin). We mapped significantly changed metabolites to KEGG compounds identifiers using MetaboAnalyst [56] and tested expected pathway utilization based on the KEGG human metabolic model [57]. In cohort 1, urine metabolites are detected to be significantly reduced after the diet in 2 pathways, *caffeine metabolism* and *histidine metabolism* (*P*_FDR_ < 0.10, hypergeometric test, Tables A and B in S8 Table), reflecting the removal of coffee and animal proteins in the controlled diets, as noted. These pathways are not observed in cohort 2 where the citrate cycle and alanine, aspartate, and glutamate metabolism pathways are enriched among the increased metabolites after the diet. These metabolites likely associate with the increase in fruits and vegetables on the vegetarian diet and suggest changes in energy utilization through citric acid cycle energy metabolism. In plasma, the caffeine metabolism pathway is significantly reduced in both cohorts and lysine degradation is reduced in the second year (*P*_FDR_ < 0.10, hypergeometric test, Tables C and D in S8 Table).

## Discussion

Disentangling precisely what shapes human microbiome variation within body sites remains challenging [2] and is a central motivation for this study. Not only does microbiome variation associate with a broad array of factors including sex, age, health status, diet, among other variables [2,16,58], but deconstructing this variation becomes increasingly complex as multiomic and/or longitudinal profiling are deployed to capture interacting or associated omic systems that may be crucial to unraveling health and disease [59–61]. Relatedly, human microbiome variation associates with many of the same diseases that are linked to health disparities in the US [62,63] including obesity [64], diabetes [64,65], colorectal cancer [66], inflammatory bowel disease [67], tuberculosis [68], and periodontitis [63]. These diseases also associate with gut and/or oral microbiomes variation [69–74]. Thus, as the ability to modulate microbiomes through social, structural, and therapeutic approaches gains interest and relevance, there is a need to carefully (i) catalog what types of multiomic and longitudinal variation reproducibly occur across ethnicities; and (ii) disentangle the complex social, cultural, and geographic factors associated with health disparities that shape system biology features.

The clinical, multiomic, and multiethnic study here builds on previous observations of ethnicity-associated microbiome variation [14–17,75] in order to test whether controlling diet among participants matched for age, BMI, sex, geography, and health status unravels the impacts in shaping interethnic, multiomic variation. Specifically, we explored changes in oral and gut microbiomes and viromes, as well as blood and urine metabolomes in response to a vegetarian diet intervention in healthy, normal weight, adult female participants who live in the same region but differ by self-reported ethnicity. Here, participants who were not previously vegetarians were provided similar vegetarian nutrient profiles that could either ablate the ethnicity differences in the microbiome or have no effect, thus uncovering whether habitual dietary differences influence ethnicity-associated microbiome variation.

We report 3 key findings: (i) ethnicity-associated variation in microbial and viral taxonomic compositions, microbial functions, and individual taxon abundances persistently occurs before, during, and after a shared, short-term diet; (ii) several previously identified heritable taxa in the gut exhibit differential abundance (DA) between ethnicities, although fewer than previously described; and (iii) in contrast to the microbiome taxonomic and functional diversity, the urine and plasma metabolomes vary with regards to the dietary intervention. Results and significance are discussed in further detail below.

### Interpersonal and ethnicity-associated variation persist during a shared diet

Interpersonal and ethnicity-associated variation persistently shape taxonomic and functional variation in gut and oral metagenomes and the purified virome during a shared dietary intervention. Among 219 abundant microbial taxa in cohort 1 and 182 abundant microbial taxa in cohort 2, 10.6% to 49.5% from the gut and 0% to 15.5% from saliva vary in abundance between ethnicities. Similarly, 13.7% of 102 genera of classified gut viruses varied between ethnicities across cohorts, comprising the first report of ethnicity-associated viromes in the US. Likewise, variation between ethnicities was observed for metagenomic functions including COGs, KO functional orthologs, ARGs discussed below, and CAZymes. Among 15 dietary intervention studies using plant-based foods, there was only a modest effect on microbial diversity in 10 of the studies, with the remaining 5 studies yielding no significant changes in microbial community composition [76]. Similar to previous work showing that switching to a new animal-based diet has been shown to alter the microbiome in as little as 2 days [28], differences in diet (before, during, and after vegetarian diet intervention) were also associated with taxonomic and functional variation in the microbiome. However, the effect size of diet was modest in our study, and, as everyone in our study received the dietary intervention, we are unable to clearly distinguish the relatively small effect of the diet from daily variation in microbiome composition and function. Results thus indicate that degrees of ethnicity-associated variation in gut and oral metagenomes, as well as gut viromes, are generally persistent through the vegetarian diet. We found consistent associations between ethnicity and ARG compositional profiles in both gut and oral microbiomes in cohort 2 (S1 Data and Fig 3).

Antibiotics and xenobiotics are important factors in structuring and altering the human gut microbiome [77]; additionally, the gut microbiome may act as a reservoir of drug resistance genes with implications for health disparities and future emergence of drug-resistant pathogens. Indeed, alterations of the gut microbiome following antibiotic exposure can drive evolution of antibiotic resistance, shifts in taxonomy or metagenomes, and/or pathogen colonization or increased replication associated with disease severity [78]. Interestingly, antibiotic use is not equal among ethnicities in the US, with White individuals estimated to have twice as many antimicrobial drug prescription fills relative to other groups [79]. There was no consistent correlation between ethnicity and antibiotic use in the study here.

There was a novel and strong association of ethnicity with CAZyme composition within a single geographic location. We identified 15 CAZyme categories that were differentially abundant in the same direction between Black and White individuals in both cohorts, and 130 KEGG pathway annotations replicated enrichment within 1 ethnicity across cohorts. Intriguingly far more KOs were enriched in 1 ethnicity or the other during (*N =* 113) the dietary intervention than before (*N* = 2) or after (*N* = 15), and these enriched pathways within 1 ethnicity represented core microbial functions most often related to amino acid metabolism and oxidative phosphorylation energy production. Differences in CAZyme and functional pathway composition and diversity have previously been reported across different geographic locations and lifestyles for both the human oral [80] and gut microbiomes [81,82]. In addition, the taxonomic composition and functional capacity of the oral microbiome are strongly influenced both by recent diet [83] and human population histories of local adaptations to specific food resources [84,85].

### Heritable taxa do not consistently associate with ethnicity

Among 9 gut taxa previously identified to be heritable based on twin and GWAS studies [50,51,86,87] and that exhibited ethnicity-associated variation in abundance [14,17,19,20], 2 were significantly different in saliva abundance in cohort 1, and 2 heritable taxa were significantly different in gut abundance in cohort 2 in this study. The Christensenellaceae family, which was heritable in cohort 2 in our study, is notable as it is the most highly heritable taxon in the human gut microbiome, and it is related to several health traits including obesity and inflammatory bowel disease [50–52]. Members of Christensenellaceae family are typically short chain fatty acids producers, which contribute to other microbes and human mucosal epithelium growth [50,88]. Ethnically varying microbial taxa previously reported to exhibit heritability may be driven by functional complementarity, play important roles in gut ecosystems, and be associated with historical population-level differences in dietary practices. There are many reasons why an inverse, abundance relationship exists between the oral and gut sites, but variation in human control of microbial abundances or induction of lytic phages due to environmental pressures could contribute. For example, heritable taxa such as *Veillonella* exhibited notable differential phage abundance between sites, such that when the bacterial genus was abundant, phage activity was lower and vice versa. With regard to human health, *Veillonella* are incapable of metabolizing common sugars and typically ferment energy sources such as lactate [89], an association that may be tied to performance in mice via metabolic conversion of exercise-induced lactic acids into propionate [90]. Taken together at the metagenomic and viromic levels across 2 body sites, a small subset of heritable taxa vary by ethnicity despite the shared dietary intervention.

The consistent community-level ethnicity-associated variation in the microbiome that persisted during the shared diet intervention suggests that there is an effect of factors other than diet that intersect with ethnicity on the microbiome, similar to the findings of other recent work examining ethnicity, diet, and gut microbiome variation [17,91,92]. Thus, several other factors that covary with or are encompassed by ethnicity could be contributing to the observed pattern of differences in gut and oral microbiomes in our study. Ethnicity can encompass differences in lifestyle, geography, and ancestry, all of which are known to contribute to microbiome variation [50,93–96]. Additionally, ethnicity intersects with other social and environmental factors known to affect the microbiome, such as socioeconomic status, exposure to environmental pollutants, and social stress [11,97–100]. While other studies have shown ethnicity-associated microbiome variation when controlling for shared geography [14,101,102], few studies have also controlled for diet. Ancestry may be contributing to some of the ethnicity-associated variation in our study, but few heritable taxa consistently differ in abundance across both cohorts. Other sources of variation, such as sociodemographic, social, cultural, and environmental factors, are likely driving the ethnicity-associated variation observed here; however, we are unable to examine these factors due to the sample size and specific characteristics of our study population.

### Metabolomic patterns reflect diet but are not distinguishable between ethnicities

A consistent trend from this work is the differential impact of the dietary intervention on microbiomes and metabolomes, whereby urine and plasma metabolome changes dominated gut and oral microbiome changes in response to the diet. Conversely, gut and oral microbiome compositional variation prevailed over metabolome differences between ethnicities. While these patterns may reflect body site-specific variation, one other explanation is that while taxa and metagenomic traits consistently vary between ethnic groups across the diet, gene expression may be more responsive to dietary input as functional reservoirs in the microbiome may be expressed in response to different inputs [103,104]. Diet-induced abundance changes of reported urine and plasma metabolites with available annotations were characterized by a shift toward metabolites from plant-based sources such as soy, garlic, and green leafy vegetables and away from animal protein, coffee, and chocolate, which validate the overall adherence to the dietary intervention. Roles of plant-based diets have been observed in multiethnic, metabolomic studies in which fecal and plasma metabolome variation associated with habitual dietary content, which differ between ethnic groups [105,106]. Taken as a whole, these results demonstrate the dietary intervention had a more prominent effect on metabolites with only limited detection of ethnicity-associated metabolite differences.

## Conclusion

Ultimately, this multiethnic, multiomic, dietary, and longitudinal study examines oral and gut metagenomes, plasma and urine metabolomes, and gut viromes in age-controlled female participants on a controlled, vegetarian diet. A major finding is that ethnicity-associated metagenomic variation in the oral and gut microbiome and gut virome persisted before, during, and after the diet, while plasma metabolomes, and to a lesser extent urine metabolomes, generally shifted with the diet and overall lacked an association with ethnicity. This study presents the first evidence of ethnicity signatures in gut viromes in a US-based population. Ethnicity-associated abundance differences in bacterial and viral taxa are also common, and a few of the differentially abundant bacterial taxa were previously deemed as heritable. Together, results represent a controlled examination of individual, ethnicity, and dietary associations in the human microbiome, virome, and metabolome. Disentangling microbiome diversity across the diversity of humans is an important imperative for potentially modulating and/or predicting social and environmental factors and microbiome compositions that might contribute to the onset and/or prevention of health disparities.

## Materials and methods

### Human participants

This study was approved by the Vanderbilt Institutional Review Board (IRB#: 171170) and has been previously reported in Materials and Methods of [46]. Briefly, informed consent was obtained from all study participants prior to any study procedures. Thirty-eight female adult participants between the ages of 18 and 40, 17 Black and 21 White individuals, enrolled in the study, and 1 Black participant and 1 White participant dropped out prior to completing the study. The authors acknowledge the complexity of defining ethnicity through racial, ancestral, social, and other constructs and use the self-reported categories of Black and White here since this work is not focused on ancestry, but rather social self-identification as non-Hispanic Black or White.

### Inclusion and exclusion criteria

Healthy women (age 18 to 40 years; BMI 18.5 to 24.9 kg/m^2^) were included in the study if they were self-declared single ethnicity (Black non-Hispanic or White non-Hispanic) for the participant and both parents. Exclusion criteria included the following: chronic disease, current illness/infection/inflammatory state, use of tobacco products, drug use, alcohol intake >2 drinks/week, currently pregnant or breastfeeding, dietary restrictions/food allergies, if they were vegetarian or vegan, use of dietary supplementation over past 3 months, or if their weight was not stable over past 3 months.

### Participant recruitment and visits

Participants were recruited from the greater area of Nashville, Tennessee, USA. All participants provided written consent forms approved by the Vanderbilt Institutional Review Board. Recruitment was carried out on local college campuses; therefore, participants are biased toward those who attend or work at a local college or university and are not necessarily representative of the broader Nashville population. Initial visits involved informed consent, medical background screening, measurements of vitals (blood pressure, pulse, respiration, temperature, height, weight), and collection of the first round of saliva, urine, and blood samples (see following sampling sections for specific protocols). At the initial visit to the Vanderbilt Center for Human Nutrition, 4 days of vegetarian food were provided (3 meals and 1 snack per day) alongside gut sampling kits, gloves, and FecesCatchers. A detailed description of the dietary intervention provided by the Vanderbilt Center for Human Nutrition has been previously reported in the Materials and methods of [46]. Participants were then asked to provide 2 days of stool samples while on their normal diet, consume the provided diet for 4 days while collecting stool samples, then return to their normal diet for 2 days with stool samples collected. At the end of this period, participants returned to the Vanderbilt Center for Human Nutrition, where the final set of oral saliva, urine, and blood samples were collected. Questionnaires were filled out to collect personal metadata using one initial pre-survey, daily surveys with each stool sample were collected, and a post-survey was conducted for participant feedback. All personal or identifiable information was stored in Vanderbilt’s secure REDCap clinical trial system (https://redcap.vanderbilt.edu/), and survey questions were approved by the Vanderbilt Institutional Review Board.

### Sampling

Study participants self-collected saliva samples using the OMNIgene Oral Kit (DNA Genotek). All samples were collected in the morning at the participants’ time of visit to the Vanderbilt Center for Human Nutrition for pre- and post-diet time points. Participants were asked to avoid tooth brushing, flossing, and use of mouthwash for 12 hours prior to sampling. Participants were asked to avoid eating, drinking, or chewing gum for 30 minutes prior to sampling. At the time of sampling, participants were asked to wash their hands and rinse their mouth with fresh water. One minute after expelling water rinse, participants spit fresh saliva into OMNIgene Oral collection funnels to the specified fill line and closed the lid to introduce stabilizing solution. Finally, collection tubes were sealed and shaken for 10 or more seconds to homogenize the sample among the stabilization solution, before being submitted to the research team for storage in −80°C freezers.

Study participants self-collected stool samples using Zymo DNA/RNA Shield Fecal Collection Tubes. Participants were instructed to collect samples from the first bowel movement of the day. Participants were asked to wash their hands and wear gloves, then place FecesCatchers across the toilet to catch stool samples. After depositing the stool on the FecesCatcher, participants were instructed to collect a small (approximately 1 gram) sample using the scoop in the DNA/RNA Shield Fecal Collection Tube, to immediately submerge in 10 mL of stabilization solution and shake by hand to mix, then to flush the FecesCatcher and remaining stool down the toilet. The collection scoop was reconnected back into the collection tube and vigorously shaken for 30 seconds to thoroughly homogenize the sample with DNA/RNA Shield solution. Samples were stored at room temperature until the post-study participant visit to the Vanderbilt Center for Human Nutrition, where all samples were returned to researchers and stored in −80°C freezers.

### Microbial metagenomics sequencing and profiling

All of the following steps of extraction and metagenomics library preparation were performed in a SterilGARD III Advance—Class II biological safety cabinet. Prior to every use, the interior of the cabinet was thoroughly cleaned with 70% ethanol and left for at least 15 minutes under UV exposure. At no point were sample tubes opened outside of the biosafety hood. An Eppendorf 24-place centrifuge (ID 5424) was thoroughly cleaned with 70% ethanol and left in the biosafety hood throughout all extractions to minimize movement in and out of the hood.

Genomic DNA from homogenized gut or oral samples were extracted with ZymoBIOMICS DNA/RNA Miniprep Kit (Cat. No. R2002) according to the manufacturer’s instructions. The DNA quality and quantity were estimated using the Nanodrop (Thermo Fisher; Waltham, MA) and Qubit (BioTek; Winooski, VT), respectively. Next, we followed the protocol for the Nextera DNA Flex Library Preparation kit (Illumina, San Diego, CA). Samples were pooled in equimolar portions and assessed using the High Sensitivity Bioanalyzer. The pooled libraries were sequenced using the Illumina NovaSeq instrument (Illumina, San Diego, CA) with 150 bp paired-end reads. Sequencing for cohort 1 produced a total of 1.9 billion reads, corresponding to an average of 12.8 million paired-end reads per sample (excluding the controls). Sequencing for cohort 2 produced a total of 2.5 billion reads, corresponding to an average of 16.5 million paired-end reads per sample (excluding the controls). Raw reads were trimmed using TrimGalore [107] with default settings. On average, 0.038% of all reads were filtered out in cohort 1 and 0.026% of all reads were filtered out in cohort 2. To remove human reads, we aligned the filtered sequences to the human reference genome hg38 using Bowtie2 [108]. In cohort 1, a mean proportion of 60.1% and 1.6% is of human origin in oral and gut metagenomes, respectively. In cohort 2, a mean proportion of 64.11% and 0.31% is of human origin in oral and gut metagenomes, respectively. A total of 332 million reads from cohort 1 and 327 million reads from cohort 2 were removed due to human DNA contamination. All gut or oral metagenomes from the same participant were coassembled respectively using MEGAHIT [109]. Assemblies with a minimum contig length of 1,000 bp from all participants were compiled as a contig database. In cohort 1, a total of 1,906,413 contigs, with an average N50 of 7,673, a maximum length of 634,712 bp and total bases of 7,480,162,758 were obtained. In cohort 2, a total of 1,954,358 contigs, with an average N50 of 7,753, a maximum length of 675,835 bp and total bases of 7,679,497,031 were obtained. Seven metagenomes in cohort 1 and 3 metagenomes in cohort 2 with less than 0.5 million reads were omitted from the downstream analysis as suggested by Hillmann and colleagues [110], resulting in 225 stool samples and 72 saliva samples. Some participants did not provide a stool sample for every day of the study. Clean reads were then mapped to the contig database using BWA [111] and SAMtools [112]. Contigs were taxonomically annotated using Kaiju [-a greedy -e 3 -E 0.00001] [113]. Contigs were also screened for COGs using Prokka [114]. For the ARGs and CAZymes, we first predicted the open reading frame of the contigs using Prodigal in metagenomic mode [115]. Subsequently, ARGs were annotated using hmmscan [—cut_ga] in HMMER v3.2.1 [47] against Resfams [48]. CAZymes were annotated using hmmscan against custom CAZyme families built from dbCAN2 and PFAM with the cutoff of e-value of 1 × 10^−15^ and coverage of 0.35 [116]. The functional abundances were normalized by the total annotated sequences.

For the validation assembly-free analysis, raw sequences were trimmed and quality-filtered using KneadData (v.0.9.0). Taxonomic and functional profiling was performed using HUMAnN (v3.0.0.alpha.4) [117]. Quality-filtered sequences were annotated with ARGs using ShortBRED [118] and the Comprehensive Antibiotic Resistance Database (CARD) (https://card.mcmaster.ca/).

### Viral metagenomics sequencing and profiling

Viral particles from 109 and 105 stool samples in cohorts 1 and 2 were purified using CsCl gradient ultracentrifugation of suspended gut material as previously described [119]. Briefly, suspension was vortexed vigorously for 10 minutes before filtration through a 0.22-μm filter. The filtrate was then loaded onto a CsCl gradient (1.3, 1.5, 1.7 g/mL) and spun at 60,000 × g for 3 hours to separate viral particles. The 1.3 to 1.5 g/mL fraction was then collected, treated with 20% vol/vol chloroform to remove bacterial vesicles, and exposed to 2 U/μL of DNase I for 3 hours at 37°C to remove free DNA from viral particles. After 3 hours, the enzymatic reaction was quenched with a final concentration of 20 mM EDTA, and DNA within viral particles was extracted using the QIAamp MinElute Virus Spin Kit (Cat. No. 57704).

The DNA quality and quantity were estimated using the Nanodrop (Thermo Fisher; Waltham, MA) and Qubit (BioTek; Winooski, VT), respectively. Next, libraries were prepared with the Nextera DNA Flex Library Preparation kit (Illumina, San Diego, CA) according to the manufacturer’s protocol. Samples were pooled in equimolar portions and assessed using the High Sensitivity Bioanalyzer. The pooled libraries were sequenced using the Illumina NovaSeq instrument (Illumina, San Diego, CA) with 250 bp paired-end reads.

Sequencing produced a total of 812,878,630 reads and 493,367,555.00, corresponding to an average of 8.9 million (±9.2 million) and 5.6 million (±5.4 million) paired-end reads per participant sample in cohorts 1 and 2, respectively. Raw reads were trimmed using Trimmomatic 0.31 [120] with default settings. To filter all human reads, all trimmed reads were mapped to the human reference genome hg38 using Bowtie2 [108]. All metagenomes from the same participant were coassembled respectively using MEGAHIT [109]. Viruses in each assembly were identified utilizing VirSorter [121], and only identified viruses within categories 1 and 2 were retained for analyses. Contigs shorter than 5,000 bp were removed from the analysis and remaining viral contigs were compiled as a contig database. Metagenomes with less than 0.5 million reads were omitted from the downstream analysis as suggested for bacteria by Hillmann and colleagues (110), resulting in 86 and 79 stool samples per cohort. Clean reads were then mapped to the de novo contig database using BowtieBatch and Read2RefMapper [122] with default settings. Contigs were taxonomically annotated using CAT [123]. Contigs were also screened for COGs using Prokka [114]. For the annotation of ARGs and peptidoglycanase, we first predicted the open reading frame of the contigs using Prodigal in metagenomic mode [115]. Subsequently, ARGs were annotated using hmmscan [—cut_ga] in HMMER v3.2.1 [47] against Resfams [48]. Peptidoglycanase was annotated using hmmscan against the CAZyme families with the cutoff of e-value of 1 × 10^−15^ and coverage of 0.35 [116].

### Microbial and viral metagenomic analysis

All data analysis was done in R (https://www.r-project.org/). The permutational multivariate analysis of variance (PERMANOVA) was performed with Bray–Curtis distance and Binary Jaccard distance on the microbiome at the strain level and the functional potential at the metagenome level using adonis2 function in *vegan* [124] with (perm = with(data, how(nperm = 999, blocks = Day)). As each individual was only sampled once per day, this model design was chosen to simultaneously control for repeated measures within an individual and dietary intervention stage. The exception to this was the model testing for the effects of individual identity and dietary intervention stage, where both terms were included in the model. The adonis2 function using marginal sums of squares (by = “margin”) was used to ensure that the order of factors in the models did not impact the proportion of the variance assigned to them. All the figures were generated in ggplot2 aside from the phylogenetic tree. The nonmetric multidimensional scaling (NMDS) ordination plots based on Bray–Curtis distance was generated to visualize the differences in microbiome and functional traits. DA analysis was performed with the LinDA function in MicrobiomeStat R package (v1.1) [125] for linear mixed-effects model: linda(taxa, meta, formula = ‘~ Ethnicity + Antibiotics Use + Hormonal Contraceptive + (1|Day); zero.handling = ‘pseudo-count’, feature.dat.type = ‘count’, prev.filter = 0, is.winsor = TRUE, outlier.pct = 0.03, p.adj.method = “BH”, alpha = 0.05).

A phylogenetic tree including abundant taxa (>1% abundance in at least 1 gut or saliva sample) was obtained from NCBI common tree. The Interactive Tree of Life [126] was used to plot the heat maps on phylogenetic tree.

### Gut microbial metagenomic KEGG functional analysis

Two-way Wilcoxon rank-sum testing with multiple test correction (*P*_FDR_ < 0.10) was performed on the relative abundances of contig-assembled KOs for each time period (before, during, and after). Significant KOs in each period were compared across cohorts to identify KOs that were significantly more abundant in the same ethnicity in both cohorts for manual annotation.

### Metabolomics analysis

Plasma and urine metabolites were commercially assayed by Metabolon (Metabolon, Morrisville, NC, USA) with their global metabolomics profiling platform, as previously described [46]. Briefly, blood and urine were fractionated and underwent reverse phase ultrahigh-performance liquid chromatography–tandem mass spectroscopy with positive and negative ion mode electrospray ionization and analysis by the coupled hydrophilic interaction chromatography. Measured compounds were then identified by Metabolon through comparison to their library of chemical standards and unknowns. A total of 796 and 842 metabolites were identified in plasma and urine samples, respectively. Raw peak intensities were quantified using the area under the curve method. Raw intensity data were median scaled and natural log transformed prior to statistical analysis. Missing intensity values were imputed at the lowest present value for each metabolite. For principal component analysis (PCA), samples were normal scaled and metabolites with zero variance were removed. R (https://www.r-project.org/) was used to perform all statistical analysis. Using *adonis* function in *vegan* package [124], PERMANOVA was used to test global and class metabolomes with 999 permutations using Euclidean distance. Tools from the MetaboAnalystR package (2.0.1) [56] were utilized in custom R scripts to perform paired nonparametric testing of metabolites (Wilcoxon rank-sum test for continuous variables) from each participant before and after the diet and to generate plots. KEGG was used to determine pathway enrichment using KEGG compound IDs associated with the measured metabolites. Ggplot2 was additionally used to generate metabolite plots. Due to the identification of outliers driving pairwise distances during the dietary analysis (Fig 5), 1 White individual was removed as an outlier in cohort 1, After Diet group. Similarly, 1 White and 1 Black individual were removed from cohort 2, After Diet analysis. Statistics in parentheses denote the removal of outlier participants in the after diet phase from each cohort based upon distances that were greater than the third quartile + 1.5 * IQR.

## Supporting information

S1 Data Numerical data underlying Figs 1B, 1C, 2A, 2B, 2C, 2D, 2E, 2F, 3, 4, 5, S2, S3, S4 and S5. (XLSX)

S2 Data Nutrients intake (kcal normalized). (XLSX)

S3 Data Summary of microbiome sequences. (XLSX)

S4 Data Permutational pairwise testing of beta dispersions with Bray–Curtis dissimilarity. (XLSX)

S5 Data KEGG orthologous pathway enrichment across ethnicities and functional annotations. (XLSX)

S6 Data CAZyme categories. (XLSX)

S7 Data Assembly-free microbiome differential abundance between ethnicities. (XLSX)

S8 Data Gut virome differential abundance between ethnicities. (XLSX)

S9 Data Significant metabolites after dietary intervention. (XLSX)

S1 Fig Recruitment poster for VMI study. Participants were recruited (*n =* 38) through the Vanderbilt Nutrition Center, with 19 participants completing sampling in 2018 and 17 participants completing sampling in 2019.
(DOCX)

S2 Fig Carbohydrate active enzymes (CAZymes) between 2 ethnicities (assembly-based analysis, unpaired Wilcoxon rank-sum test, FDR method for multiple test correction). (A) Gut microbiome CAZymes in cohort 1. (B) Oral microbiome CAZymes in cohort 1. (C) Gut microbiome CAZymes in cohort 2. (D) Oral microbiome CAZymes in cohort 2. (E) Gut virome peptidoglycanase in cohort 1. (F) Gut virome peptidoglycanase in cohort 2. Data underlying this figure can be found in S1 Data.
(DOCX)

S3 Fig Association between oral and gut core taxa in cohort 1 (219 taxa at or above the genus level). Triangles denote taxa that are more abundant in saliva (*n* = 69); circles denote taxa that are more abundant in gut (*n* = 150). r and *p*-values are based on Spearman correlation between the average abundance across all samples of 219 oral and gut taxa. Data underlying this figure can be found at S1 Data.
(DOCX)

S4 Fig Plasma metabolomes changed significantly as a result of the diet, while urine metabolomes are unchanged. PCA of normal scaled metabolites in urine and plasma with 95% confidence intervals in the shaded areas. Statistics are based on multivariable permutational analyses of variance (PERMANOVA) with Euclidean distance (with stage as individual factor, permutations = 999). There are no urine metabolome differences between ethnicities or with regard to diet. Plasma metabolomes in both years reflect dietary intervention among all participant’s total metabolite compositions with no difference between ethnicities. Data underlying this figure can be found at S1 Data.
(DOCX)

S5 Fig Plasma and urine metabolites do not significantly differ between ethnicities before or after the diet. Volcano plots comparing metabolites from Black:White individuals before and after diet as measured in urine and plasma. Vertical dashed lines indicate a fold-change (FC) of 1. Horizontal dashed line indicated statistical significance following nonparametric Wilcoxon rank sum test, FDR <0.05. Data underlying this figure can be found at S1 Data.
(DOCX)

S1 Table Distribution of participants, Social Vulnerability Index scores, microbial metagenomic samples, and nutritional profiles of habitual and study diets. (A) Baseline participant differences between groups and cohorts. (B) Social Vulnerability Index scores between ethnicities and individuals. (C) Distribution of participants and microbial metagenomic samples. (D) Variation attributed to differences in nutritional profiles of habitual and study diets.
(DOCX)

S2 Table Individuality accounts for most microbiome variation. (A) Assembly-based taxonomy; (B) assembly-free taxonomy; (C) assembly-based COGs categories; (D) assembly-free COGs composition; (E) assembly-based ARGs categories; (F) assembly-free ARGs compositions; (G) assembly-based CAZymes categories (peptidoglycanase for gut virome); (H) assembly-based KEGG composition; (I) assembly-free KEGG compositions; (J) intragroup differences between stages of the diet using permutational pairwise testing of beta dispersions from Bray–Curtis dissimilarities; (K) intergroup differences between stages of the diet using permutational pairwise testing of beta dispersions from Bray–Curtis dissimilarities.
(DOCX)

S3 Table Factors associated with gut and oral microbiomes in both cohorts from assembly-free analysis. (DOCX)

S4 Table Multivariate homogeneity of groups dispersion results for gut and oral microbiomes gut viromes. (A) Cohort 1 from assembly-based analysis; (B) cohort 2 from assembly-based analysis; (C) cohort 1 from assembly-free analysis; (D) cohort 2 from assembly-free analysis; (E) both cohorts from assembly-free analysis.
(DOCX)

S5 Table Alpha diversity (Shannon diversity index) is stable in response to diet intervention in both cohorts based on (A) assembly-based analysis and (B) assembly-free data.
(DOCX)

S6 Table Contrasting ethnic difference of the heritable taxa in oral and gut microbiome among 2 ethnicities across the dietary intervention (assembly-based analysis, FDR <0.05, LinDA). (DOCX)

S7 Table Urine (A) and plasma (B) super pathway analysis by ethnicity.
(DOCX)

S8 Table Metabolome pathway analysis. (A) Cohort 1 urine; (B) cohort 2 urine; (C) cohort 1 plasma; (D) cohort 2 plasma.
(DOCX)

## Abbreviations

ARG: antibiotic-resistant gene
BMI: body mass index
CAZyme: carbohydrate active enzyme
COG: cluster of orthologous groups
CRP: C-reactive protein
DA: differential abundance
KO: KEGG ortholog
NDSR: Nutrition Data System for Research
NMDS: nonmetric multidimensional scaling
PCA: principal component analysis
PERMANOVA: permutational multivariate analysis of variance
SVI: Social Vulnerability Index
VMIC: Vanderbilt Microbiome Innovation Center
