# Supplementary material for: Individuality and ethnicity eclipse a short-term dietary intervention in shaping microbiomes and viromes
Source: PLoS Biol. 2022 Aug 23;20(8):e3001758. doi: 10.1371/journal.pbio.3001758 (PMC9397868; doi:10.1371/journal.pbio.3001758)
Supplement: S1 Fig — Participants were recruited (n = 38) through the Vanderbilt Nutrition Center, with 19 participants completing sampling in 2018 and 17 participants completing sampling in 2019. (DOCX) [file pbio.3001758.s010.docx]

**S1 Fig.** **Recruitment poster for VMI study**. Participants were recruited (n = 38) through the Vanderbilt Nutrition Center, with 19 subjects completing sampling in 2018 and 17 subjects completing sampling in 2019.
