# Supplementary material for: Individuality and ethnicity eclipse a short-term dietary intervention in shaping microbiomes and viromes
Source: PLoS Biol. 2022 Aug 23;20(8):e3001758. doi: 10.1371/journal.pbio.3001758 (PMC9397868; doi:10.1371/journal.pbio.3001758)
Supplement: S2 Fig — (A) Gut microbiome CAZymes in cohort 1. (B) Oral microbiome CAZymes in cohort 1. (C) Gut microbiome CAZymes in cohort 2. (D) Oral microbiome CAZymes in cohort 2. (E) Gut virome peptidoglycanase in cohort 1. (F) Gut virome peptidoglycanase in cohort 2. Data underlying this figure can be found in S1 Data. (DOCX) [file pbio.3001758.s011.docx]

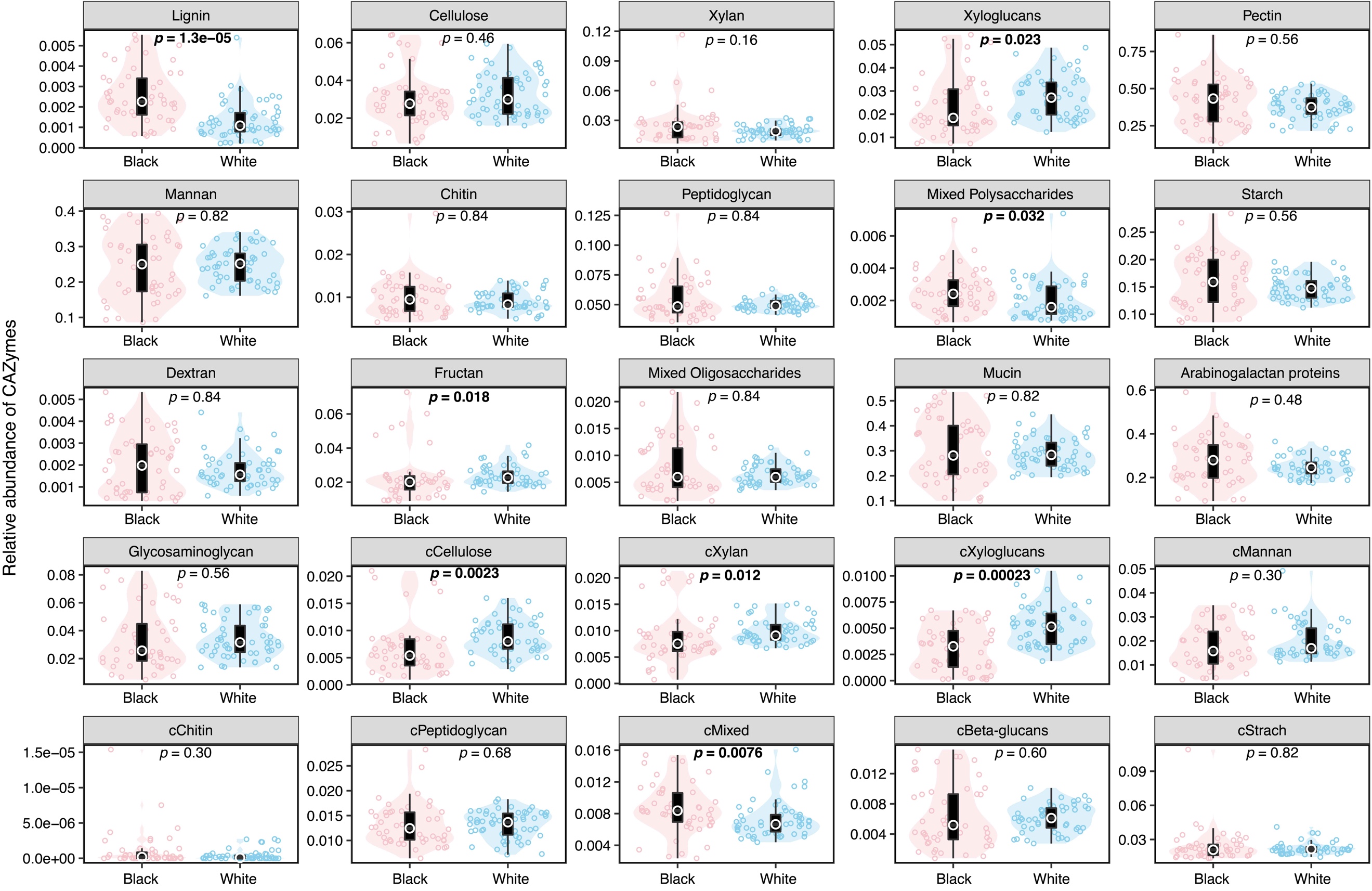


**S2A Fig.** **Gut microbiome carbohydrate active enzymes (CAZymes) between two ethnicities in cohort 1** (assembly-based analysis, unpaired Wilcoxon rank-sum test, FDR method for multiple test correction). (Data underlying this figure can be found at S1 Data)


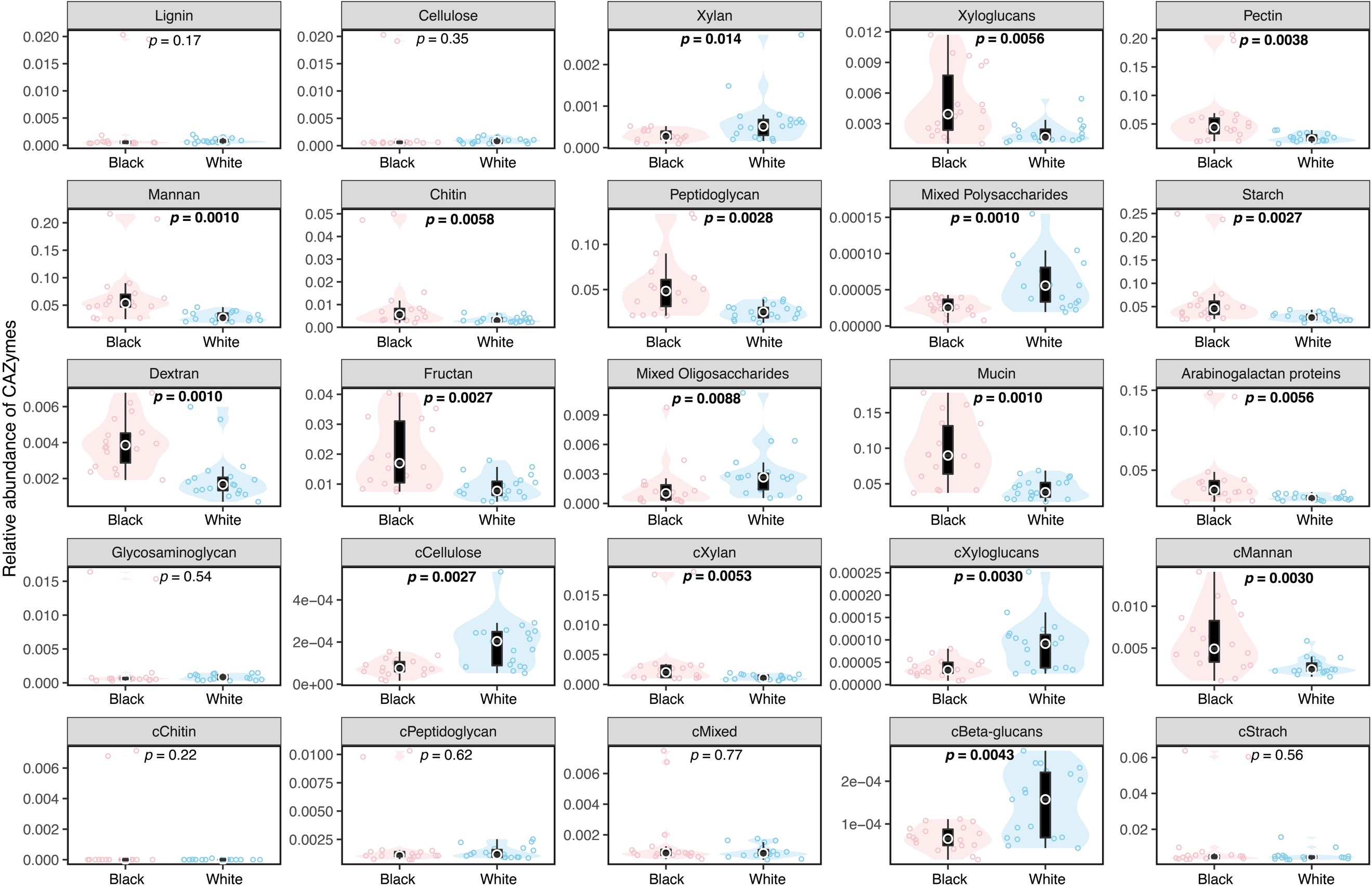


**S2B Fig.** **Oral microbiome carbohydrate active enzymes (CAZymes) between two ethnicities in cohort 1** (assembly-based analysis, unpaired Wilcoxon rank-sum test, FDR method for multiple test correction). (Data underlying this figure can be found at S1 Data)


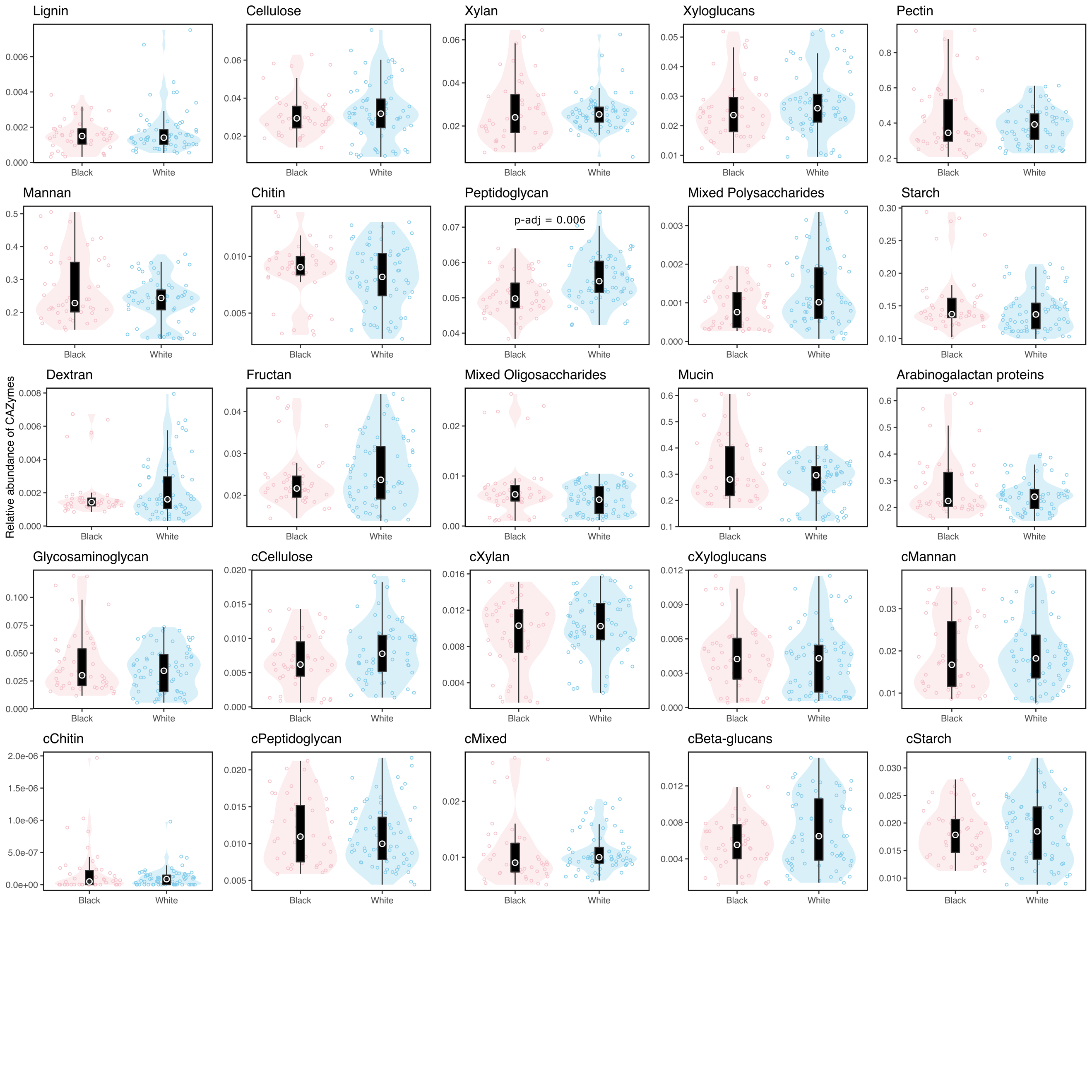


**S2C Fig.** **Gut microbiome carbohydrate active enzymes (CAZymes) between two ethnicities in cohort 2** (assembly-based analysis, unpaired Wilcoxon rank-sum test, FDR method for multiple test correction). (Data underlying this figure can be found at S1 Data)


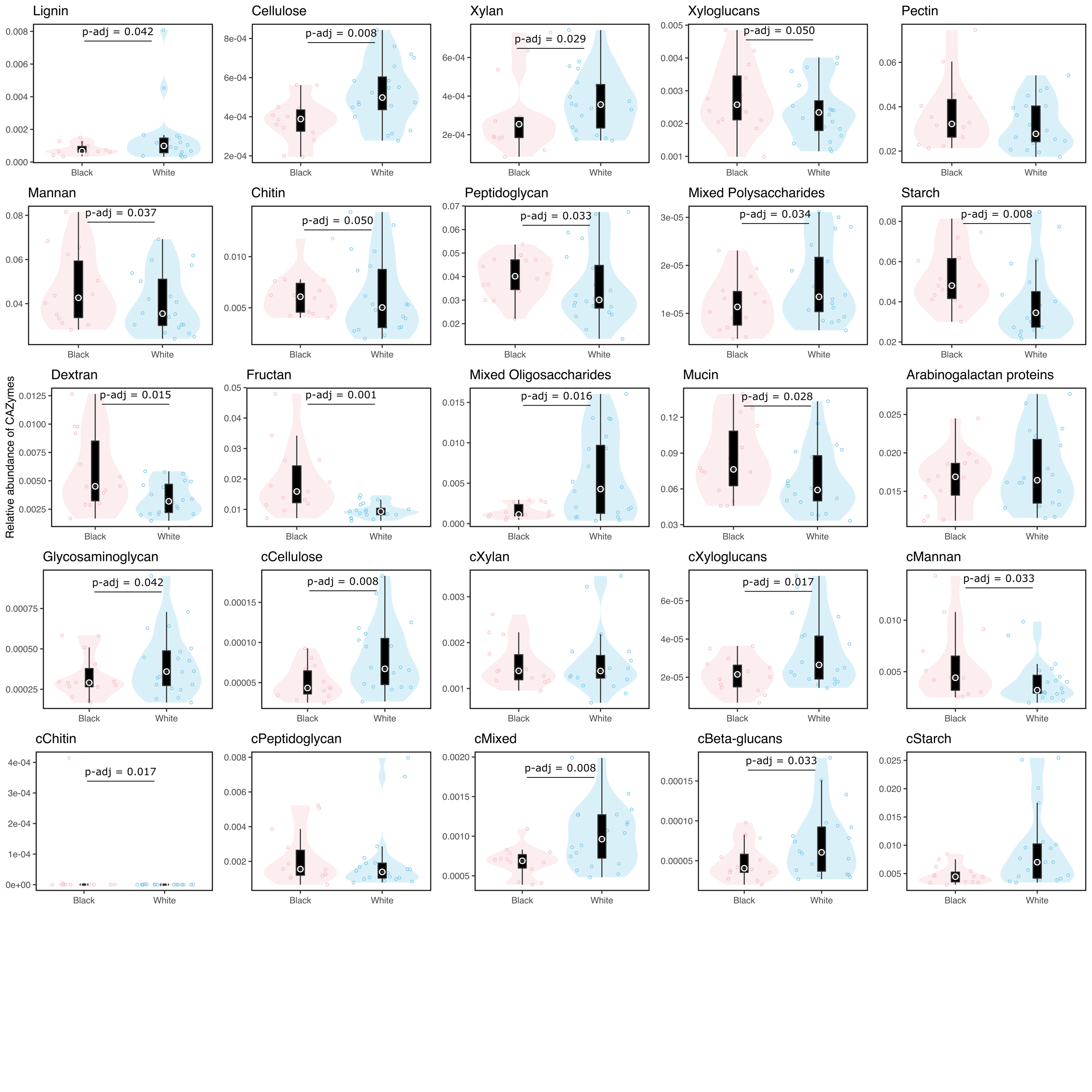


**S2D Fig.** **Oral microbiome carbohydrate active enzymes (CAZymes) between two ethnicities in cohort 2** (assembly-based analysis, unpaired Wilcoxon rank-sum test, FDR method for multiple test correction). (Data underlying this figure can be found at S1 Data)

**S2E Fig.** **Gut virome peptidoglycanase between two ethnicities in cohort 1** (assembly-based analysis, unpaired Wilcoxon rank-sum test, FDR method for multiple test correction). (Data underlying this figure can be found at S1 Data)

**S2F Fig.** **Gut virome peptidoglycanase between two ethnicities in cohort 2** (assembly-based analysis, unpaired Wilcoxon rank-sum test, FDR method for multiple test correction). (Data underlying this figure can be found at S1 Data)
