# Supplementary material for: Individuality and ethnicity eclipse a short-term dietary intervention in shaping microbiomes and viromes
Source: PLoS Biol. 2022 Aug 23;20(8):e3001758. doi: 10.1371/journal.pbio.3001758 (PMC9397868; doi:10.1371/journal.pbio.3001758)
Supplement: S3 Fig — Triangles denote taxa that are more abundant in saliva (n = 69); circles denote taxa that are more abundant in gut (n = 150). r and p-values are based on Spearman correlation between the average abundance across all samples of 219 oral and gut taxa. Data underlying this figure can be found at S1 Data. (DOCX) [file pbio.3001758.s012.docx]

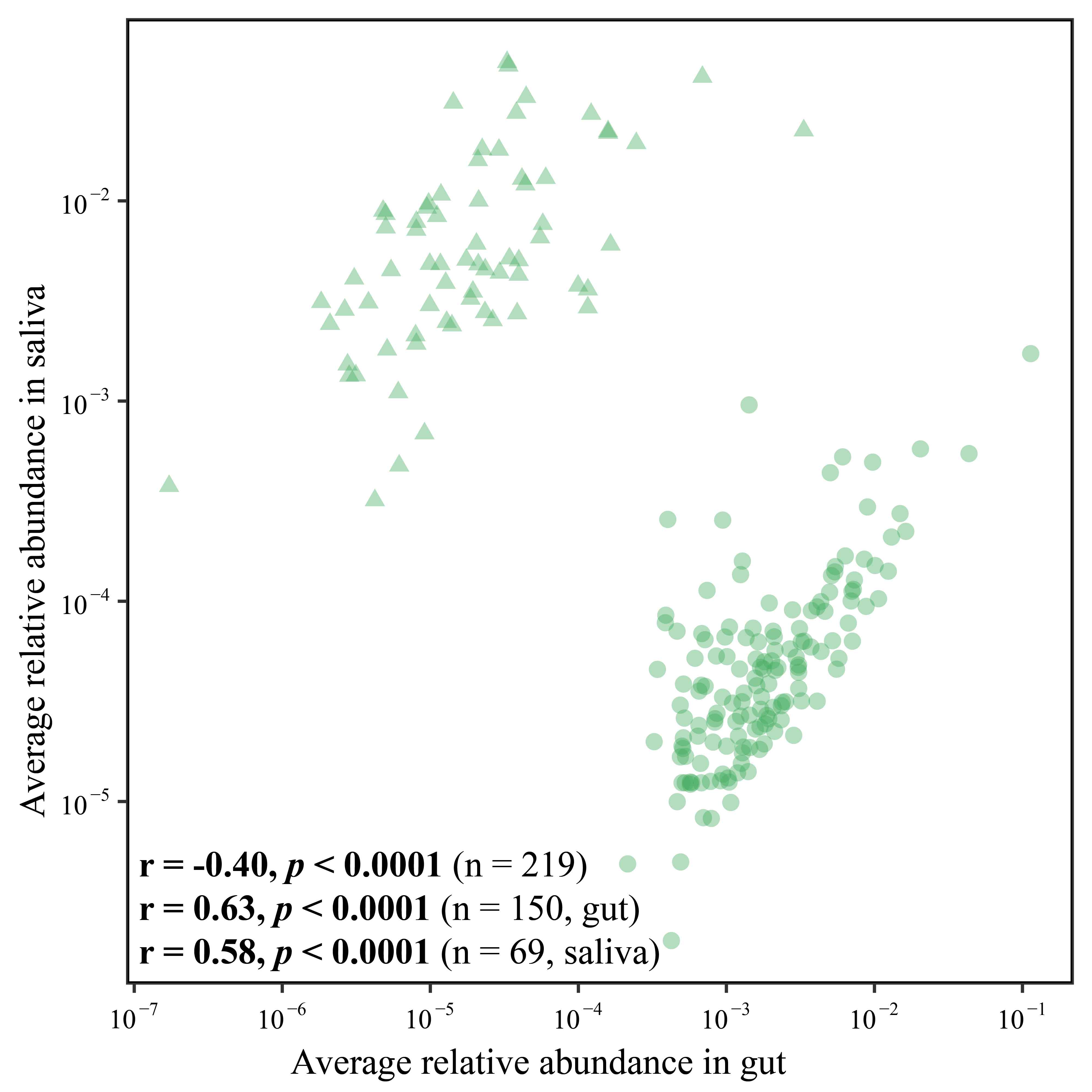


**S3 Fig. Association between oral and gut core taxa in cohort 1 (219 taxa at or above the genus level).** Triangles denote taxa which are more abundant in saliva (n = 69); Circles denote taxa which are more abundant in gut (n = 150). r and *p* values are based on Spearman correlation between the average abundance across all samples of 219 oral and gut taxa. (Data underlying this figure can be found at S1 Data)
