# Supplementary material for: Individuality and ethnicity eclipse a short-term dietary intervention in shaping microbiomes and viromes
Source: PLoS Biol. 2022 Aug 23;20(8):e3001758. doi: 10.1371/journal.pbio.3001758 (PMC9397868; doi:10.1371/journal.pbio.3001758)
Supplement: S4 Fig — PCA of normal scaled metabolites in urine and plasma with 95% confidence intervals in the shaded areas. Statistics are based on multivariable permutational analyses of variance (PERMANOVA) with Euclidean distance (with stage as individual factor, permutations = 999). There are no urine metabolome differences between ethnicities or with regard to diet. Plasma metabolomes in both years reflect dietary intervention among all participant’s total metabolite compositions with no difference between ethnicities. Data underlying this figure can be found at S1 Data. (DOCX) [file pbio.3001758.s013.docx]

**S4 Fig. Plasma metabolomes changed significantly as a result of the diet, while urine metabolomes are unchanged**. PCA of normal scaled metabolites in urine and plasma with 95% confidence intervals in the shaded areas. Statistics are based on multivariable permutational analyses of variance (PERMANOVA) with Euclidean distance (with stage as individual factor, permutations = 999). There are no urine metabolome differences between ethnicities or with regard to diet. Plasma metabolomes in both years reflect dietary intervention among all subject’s total metabolite compositions with no difference between ethnicities. (Data underlying this figure can be found at S1 Data)
