# Supplementary material for: Individuality and ethnicity eclipse a short-term dietary intervention in shaping microbiomes and viromes
Source: PLoS Biol. 2022 Aug 23;20(8):e3001758. doi: 10.1371/journal.pbio.3001758 (PMC9397868; doi:10.1371/journal.pbio.3001758)
Supplement: S5 Fig — Volcano plots comparing metabolites from Black:White individuals before and after diet as measured in urine and plasma. Vertical dashed lines indicate a fold-change (FC) of 1. Horizontal dashed line indicated statistical significance following nonparametric Wilcoxon rank sum test, FDR <0.05. Data underlying this figure can be found at S1 Data. (DOCX) [file pbio.3001758.s014.docx]

**S5 Fig. Plasma and urine metabolites do not significantly differ between ethnicities before or after the diet.** Volcano plots comparing metabolites from Black:White individuals before and after diet as measured in urine and plasma. Vertical dashed lines indicate a fold-change (FC) of 1. Horizontal dashed line indicated statistical significance following non-parametric Wilcoxon Rank Sum Test, FDR <0.05. (Data underlying this figure can be found at S1 Data)
