# Supplementary material for: Individuality and ethnicity eclipse a short-term dietary intervention in shaping microbiomes and viromes
Source: PLoS Biol. 2022 Aug 23;20(8):e3001758. doi: 10.1371/journal.pbio.3001758 (PMC9397868; doi:10.1371/journal.pbio.3001758)
Supplement: S1 Table — (A) Baseline participant differences between groups and cohorts. (B) Social Vulnerability Index scores between ethnicities and individuals. (C) Distribution of participants and microbial metagenomic samples. (D) Variation attributed to differences in nutritional profiles of habitual and study diets. (DOCX) [file pbio.3001758.s015.docx]

**S1 Table. Distribution of subjects, Social Vulnerability Index Scores, microbial metagenomic samples, and nutritional profiles of habitual and study diets.**

**A.** **Baseline subject differences between groups and cohorts**

|  | **Cohort 1** | | | **Cohort 2** | | | **Black**  **Cohort1:Cohort2** | **White**  **Cohort1:Cohort2** |
| --- | --- | --- | --- | --- | --- | --- | --- | --- |
|  | **Black** | **White** | *P* Value | **Black** | **White** | *P* Value | *P* Value | *P* Value |
| Age (y) | 24.8 (2.7) | 27.4 (7.3) | 0.566 | 23.7 (5) | 26.1 (5.5) | 0.303 | 0.490 | 0.733 |
| BMI (kg/m^2^) | 23.1 (2.2) | 22 (1.9) | 0.236 | 22.7 (1.6) | 21.7 (2) | 0.536 | 0.470 | 0.967 |
| CRP (mg/dL) | 1.1 (1.5) | 1.9 (1.9) | 0.27 | 1.6 (1.9) | 1.4 (1.1) | 1 | 0.383 | 0.796 |

Mean (Standard Deviation) of subjects of each ethnicity within and between cohort. P-value determined by Mann-Whitney U Test for unpaired two sample means.

**B.** **Social Vulnerability Index Scores Between Ethnicities and Individuals**

|  | Df | R^2^ | F | P-value |
| --- | --- | --- | --- | --- |
| Ethnicity | 1 | 0.00388025 | 0.0787174 | 0.887 |
| Study ID | 36 | 0.40459842 | 0.2279988 | 1 |

Multivariable permutational analysis of variance (PERMANOVA) using Bray-Curtis dissimilarity on SVI Themes 1-4 with adonis2 (data ~ Ethnicity + Study_ID, data = meta, margin =T, permutations = perm, method = distance, na.rm = T); perm <- how(nperm = 999); setBlocks(perm) = with(meta,Cohort).

**C.** **Distribution of subjects and microbial metagenomic samples**

|  |  |  |  | Stool | |  | Saliva | |
| --- | --- | --- | --- | --- | --- | --- | --- | --- |
|  |  |  |  | Subject# | Sample# |  | Subject# | Sample# |
| Cohort 1 | Black | Total |  | 9 | 50 |  | 9 | 18 |
|  |  | Stage | Before | 9 | 13 |  | 9 | 9 |
|  |  |  | During | 9 | 21 |  | NA | NA |
|  |  |  | After | 9 | 16 |  | 9 | 9 |
|  |  | Hormonal contraceptive | Yes | 4 | 23 |  | 4 | 8 |
|  |  |  | No | 5 | 27 |  | 5 | 10 |
|  |  | Antibiotic use within the past year | Yes | 3 | 19 |  | 3 | 6 |
|  |  |  | No | 6 | 31 |  | 6 | 12 |
|  | White | Total |  | 10 | 55 |  | 10 | 19 |
|  |  | Stage | Before | 10 | 16 |  | 9 | 9 |
|  |  |  | During | 10 | 24 |  | NA | NA |
|  |  |  | After | 10 | 15 |  | 10 | 10 |
|  |  | Hormonal contraceptive | Yes | 4* | 22 |  | 4 | 7 |
|  |  |  | No | 6 | 33 |  | 6 | 12 |
|  |  | Antibiotic use within the past year | Yes | 6* | 31 |  | 6 | 11 |
|  |  |  | No | 4 | 24 |  | 4 | 8 |
| Cohort 2 | Black | Total |  | 7 | 46 |  | 7 | 14 |
|  |  | Stage | Before | 7 | 8 |  | 7 | 7 |
|  |  |  | During | 7 | 26 |  | NA | NA |
|  |  |  | After | 7 | 12 |  | 7 | 7 |
|  |  | Hormonal | Yes | 4* | 8 |  | 4 | 8 |
|  |  | contraceptive | No | 3 | 28 |  | 3 | 6 |
|  |  | Antibiotic use | Yes | 2* | 10 |  | 2 | 4 |
|  |  | within the past year | No | 5 | 36 |  | 5 | 10 |
|  | White | Total |  | 10 | 63 |  | 10 | 20 |
|  |  | Stage | Before | 9 | 9 |  | 10 | 10 |
|  |  |  | During | 10 | 35 |  | NA | NA |
|  |  |  | After | 10 | 19 |  | 10 | 10 |
|  |  | Hormonal | Yes | 6* | 47 |  | 6 | 14 |
|  |  | contraceptive | No | 4 | 16 |  | 4 | 6 |
|  |  | Antibiotic use | Yes | 4* | 26 |  | 4 | 8 |
|  |  | within the past year | No | 6 | 37 |  | 6 | 12 |

* Five White subjects and two Black subjects are on both hormonal contraceptive and have a history of antibiotic use within the past year; NA denotes no saliva samples were collected on the study diet

**D.** **Variation attributed to differences in nutritional profiles of habitual and study diets.**

| **Cohort One** | **R^2^** | **P** | **Cohort Two** | **R^2^** | **P** |
| --- | --- | --- | --- | --- | --- |
| Ethnicity-Habitual | 1.4% | 0.127 | Ethnicity-Habitual | 1.4% | 0.934 |
| Study Diet | 81.0% | 0.001 | All Days | 81.8% | 0.001 |
| Habitual-Day 1 | 11.2% | 0.001 | Habitual-Day 1 | 20.5% | 0.001 |
| Habitual-Day 2 | 4.7% | 0.182 | Habitual-Day 2 | 13.3% | 0.002 |
| Habitual-Day 3 | 73.9% | 0.001 | Habitual-Day 3 | 74.9% | 0.001 |
| Habitual-Day 4 | 19.7% | 0.001 | Habitual-Day 4 | 36.4% | 0.001 |

Multivariable permutational analysis of variance (PERMANOVA) using Euclidean distances on total nutritional profiles of each day of diet.
