# Supplementary material for: Individuality and ethnicity eclipse a short-term dietary intervention in shaping microbiomes and viromes
Source: PLoS Biol. 2022 Aug 23;20(8):e3001758. doi: 10.1371/journal.pbio.3001758 (PMC9397868; doi:10.1371/journal.pbio.3001758)
Supplement: S2 Table — (A) Assembly-based taxonomy; (B) assembly-free taxonomy; (C) assembly-based COGs categories; (D) assembly-free COGs composition; (E) assembly-based ARGs categories; (F) assembly-free ARGs compositions; (G) assembly-based CAZymes categories (peptidoglycanase for gut virome); (H) assembly-based KEGG composition; (I) assembly-free KEGG compositions; (J) intragroup differences between stages of the diet using permutational pairwise testing of beta dispersions from Bray–Curtis dissimilarities; (K) intergroup differences between stages of the diet using permutational pairwise testing of beta dispersions from Bray–Curtis dissimilarities. (DOCX) [file pbio.3001758.s016.docx]

**S2 Table. Individuality accounts for most microbiome variation.** A. Assembly-based taxonomy; B. assembly-free taxonomy; C. Assembly-based COGs categories; D. Assembly-free COGs composition; E. Assembly-based ARGs categories; F. Assembly-free ARGs compositions; G. Assembly-based CAZymes categories (peptidoglycanase for gut virome); H. Assembly-based KEGG composition; I. Assembly-free KEGG compositions; J. Intragroup differences between stages of the diet using permutational pairwise testing of beta dispersions from Bray-Curtis dissimilarities; K. Intergroup differences between stages of the diet using permutational pairwise testing of beta dispersions from Bray-Curtis dissimilarities.

**A.** **Individuality accounts for most variation in metagenomic communities from assembly-based analysis**

|  |  | Gut microbiome | | Oral microbiome | | Gut virome | |
| --- | --- | --- | --- | --- | --- | --- | --- |
|  |  | R^2^ | *P* | R^2^ | *P* | R^2^ | *P* |
| **Bray-Curtis** |  |  |  |  |  |  |  |
| Cohort 1 | Subject | **0.866** | **<0.001** | **0.869** | **<0.001** | **0.710** | **<0.001** |
|  | Stage | **0.006** | **<0.001** | 0.0086 | 0.296 | 0.008 | 0.669 |
| Cohort 2 | Subject | **0.856** | **<0.001** | **0.852** | **<0.001** | **0.888** | **<0.001** |
|  | Stage | 0.005 | 0.070 | 0.013 | 0.127 | 0.004 | 0.102 |
| **Binary Jaccard** |  |  |  |  |  |  |  |
| Cohort 1 | Subject | **0.564** | **<0.001** | **0.633** | **<0.001** | **0.621** | **<0.001** |
|  | Stage | **0.017** | **0.008** | 0.018 | 0.822 | 0.010 | 0.780 |
| Cohort 2 | Subject | **0.609** | **<0.001** | **0.686** | **<0.001** | **0.736** | **<0.001** |
|  | Stage | 0.011 | 0.129 | 0.020 | 0.216 | 0.008 | 0.554 |

adonis model 2 (data ~ Subject + Stage, permutations = 999, method="bray", by="margin")

**B. Individuality accounts for most variation in metagenomic communities from assembly-free analysis**

|  |  | Gut microbiome | | Oral microbiome | |
| --- | --- | --- | --- | --- | --- |
|  |  | R^2^ | *P* | R^2^ | *P* |
| **Bray-Curtis** |  |  |  |  |  |
| Cohort 1 | Subject | **0.879** | **<0.001** | **0.853** | **<0.001** |
|  | Stage | **0.006** | **<0.001** | 0.007 | 0.605 |
| Cohort 2 | Subject | **0.863** | **<0.001** | **0.848** | **<0.001** |
|  | Stage | **0.006** | **0.028** | 0.009 | 0.450 |
| **Binary Jaccard** |  |  |  |  |  |
| Cohort 1 | Subject | **0.873** | **<0.001** | **0.819** | **<0.001** |
|  | Stage | **0.007** | **0.002** | 0.012 | 0.277 |
| Cohort 2 | Subject | **0.892** | **<0.001** | **0.848** | **<0.001** |
|  | Stage | **0.005** | **<0.001** | 0.010 | 0.248 |

adonis model 2 (data ~ Subject + Stage, permutations = 999, method="bray", by="margin").

**C.** **Individuality accounts for most variation in metagenomic COGs categories from assembly-based analysis**

|  |  | Gut microbiome | | Oral microbiome | | Gut virome | |
| --- | --- | --- | --- | --- | --- | --- | --- |
|  |  | R^2^ | *P* | R^2^ | *P* | R^2^ | *P* |
| **Bray-Curtis** |  |  |  |  |  |  |  |
| Cohort 1 | Subject | **0.844** | **<0.001** | **0.953** | **<0.001** | **0.627** | **<0.001** |
|  | Stage | **0.017** | **0.006** | **<0.001** | 0.740 | 0.010 | 0.556 |
| Cohort 2 | Subject | **0.778** | **<0.001** | **0.924** | **<0.001** | **0.833** | **<0.001** |
|  | Stage | 0.004 | 0.424 | **0.019** | **0.028** | 0.006 | 0.219 |
| **Binary Jaccard** |  |  |  |  |  |  |  |
| Cohort 1 | Subject | NA | NA | NA | NA | **0.456** | **<0.001** |
|  | Stage | NA | NA | NA | NA | 0.009 | 0.791 |
| Cohort 2 | Subject | NA | NA | NA | NA | **0.640** | **<0.001** |
|  | Stage | NA | NA | NA | NA | 0.014 | 0.308 |

NA denotes all functional categories of COGs are present in gut and oral microbiomes.

adonis model 2 (data ~ Subject + Stage, permutations = 999, method="bray", by="margin").

**D. Individuality accounts for most variation in metagenomic COGs composition from assembly-free analysis**

|  |  | Gut microbiome | | Oral microbiome | |
| --- | --- | --- | --- | --- | --- |
|  |  | R^2^ | *P* | R^2^ | *P* |
| **Bray-Curtis** |  |  |  |  |  |
| Cohort 1 | Subject | **0.800** | **<0.001** | **0.859** | **<0.001** |
|  | Stage | **0.007** | **0.026** | 0.006 | 0.684 |
| Cohort 2 | Subject | **0.805** | **<0.001** | **0.797** | **<0.001** |
|  | Stage | **0.007** | **0.042** | 0.013 | 0.349 |
| **Binary Jaccard** |  |  |  |  |  |
| Cohort 1 | Subject | **0.725** | **<0.001** | **0.710** | **<0.001** |
|  | Stage | **0.012** | **0.003** | 0.014 | 0.580 |
| Cohort 2 | Subject | **0.802** | **<0.001** | **0.749** | **<0.001** |
|  | Stage | **0.006** | **0.034** | 0.023 | 0.058 |

adonis model 2 (data ~ Subject + Stage, permutations = 999, method="bray", by="margin")

**E.** **Individuality accounts for most variation in metagenomic ARGs categories from assembly-based analysis**

|  |  | Gut microbiome | | Oral microbiome | | Gut virome | |
| --- | --- | --- | --- | --- | --- | --- | --- |
|  |  | R^2^ | *P* | R^2^ | *P* | R^2^ | *P* |
| **Bray-Curtis** |  |  |  |  |  |  |  |
| Cohort 1 | Subject | **0.823** | **<0.001** | **0.963** | **<0.001** | **0.653** | **<0.001** |
|  | Stage | **0.021** | **0.004** | **0.001** | 0.665 | 0.003 | 0.969 |
| Cohort 2 | Subject | **0.798** | **<0.001** | **0.925** | **<0.001** | **0.798** | **<0.001** |
|  | Stage | **0.005** | 0.309 | **0.019** | **0.026** | 0.004 | 0.766 |
| **Binary Jaccard** |  |  |  |  |  |  |  |
| Cohort 1 | Subject | **0.506** | **<0.001** | 0.636 | 0.102 | **0.602** | **<0.001** |
|  | Stage | 0.004 | 0.748 | 0.015 | 0.483 | 0.010 | 0.535 |
| Cohort 2 | Subject | NA | NA | 0.660 | 0.106 | **0.684** | **<0.001** |
|  | Stage | NA | NA | 0.020 | 0.298 | 0.006 | 0.723 |

NA denotes all functional categories of COGs are present in gut and oral microbiomes.

adonis model 2 (data ~ Subject + Stage, permutations = 999, method="bray", by="margin").

**F. Individuality accounts for most variation in metagenomic ARGs compositions from assembly-free analysis**

|  |  | Gut microbiome | | Oral microbiome | |
| --- | --- | --- | --- | --- | --- |
|  |  | R^2^ | *P* | R^2^ | *P* |
| **Bray-Curtis** |  |  |  |  |  |
| Cohort 1 | Subject | **0.798** | **<0.001** | **0.886** | **<0.001** |
|  | Stage | 0.006 | 0.160 | 0.005 | 0.640 |
| Cohort 2 | Subject | **0.862** | **<0.001** | **0.844** | **<0.001** |
|  | Stage | 0.003 | 0.530 | 0.008 | 0.450 |
| **Binary Jaccard** |  |  |  |  |  |
| Cohort 1 | Subject | **0.579** | **<0.001** | **0.720** | **<0.001** |
|  | Stage | 0.016 | 0.068 | 0.018 | 0.343 |
| Cohort 2 | Subject | **0.544** | **<0.001** | **0.730** | **<0.001** |
|  | Stage | **0.016** | **0.019** | **0.027** | **0.033** |

adonis model 2 (data ~ Subject + Stage, permutations = 999, method="bray", by="margin")

**G.** **Individuality accounts for most variation in metagenomic CAZymes categories (peptidoglycanase for gut virome) from assembly-based analysis**

|  |  | Gut microbiome | | Oral microbiome | | Gut virome | |
| --- | --- | --- | --- | --- | --- | --- | --- |
|  |  | R^2^ | *P* | R^2^ | *P* | R^2^ | *P* |
| **Bray-Curtis** |  |  |  |  |  |  |  |
| Cohort 1 | Subject | **0.864** | **<0.001** | **0.962** | **<0.001** | **0.603** | **<0.001** |
|  | Stage | 0.003 | 0.431 | 0.003 | 0.289 | 0.005 | 0.956 |
| Cohort 2 | Subject | **0.854** | **<0.001** | **0.871** | **<0.001** | **0.888** | **<0.001** |
|  | Stage | **0.008** | 0.066 | 0.016 | 0.118 | 0.004 | 0.347 |
| **Binary Jaccard** |  |  |  |  |  |  |  |
| Cohort 1 | Subject | **0.290** | **0.026** | **0.407** | **0.721** | **0.523** | **<0.001** |
|  | Stage | 0.014 | 0.427 | 0.022 | 0.596 | 0.009 | 0.742 |
| Cohort 2 | Subject | **0.275** | **0.009** | **0.514** | **0.608** | **0.679** | **<0.001** |
|  | Stage | 0.047 | 0.052 | 0 | 1.0 | 0.007 | 0.718 |

adonis model 2 (data ~ Subject + Stage, permutations = 999, method="bray", by="margin")

**H.** **Individuality accounts for most variation in metagenomic KEGG composition from assembly-based analysis**

|  |  | Gut microbiome | | Oral microbiome | |
| --- | --- | --- | --- | --- | --- |
|  |  | R^2^ | *P* | R^2^ | *P* |
| **Bray-Curtis** |  |  |  |  |  |
| Cohort 1 | Subject | **0.819** | **<0.001** | NA | NA |
|  | Stage | **0.007** | **0.023** | NA | NA |
| Cohort 2 | Subject | **0.817** | **<0.001** | NA | NA |
|  | Stage | **0.004** | 0.336 | NA | NA |
| **Binary Jaccard** |  |  |  |  |  |
| Cohort 1 | Subject | **0.989** | **<0.001** | NA | NA |
|  | Stage | **0.0003** | **0.046** | NA | NA |
| Cohort 2 | Subject | **0.578** | **<0.001** | NA | NA |
|  | Stage | 0.010 | 0.389 | NA | NA |

NA denotes KEGG were not measured in oral microbiomes. Bold numbers are below P < 0.05.

adonis model 2 (data ~ Subject + Stage, permutations = 999, method="bray", by="margin").

**I. Individuality accounts for most variation in metagenomic KEGG compositions from assembly-free analysis**

|  |  | Gut microbiome | | Oral microbiome | |
| --- | --- | --- | --- | --- | --- |
|  |  | R^2^ | *P* | R^2^ | *P* |
| **Bray-Curtis** |  |  |  |  |  |
| Cohort 1 | Subject | **0.788** | **<0.001** | **0.863** | **<0.001** |
|  | Stage | 0.008 | 0.056 | 0.006 | 0.596 |
| Cohort 2 | Subject | **0.819** | **<0.001** | **0.783** | **<0.001** |
|  | Stage | 0.005 | 0.216 | 0.017 | 0.227 |
| **Binary Jaccard** |  |  |  |  |  |
| Cohort 1 | Subject | **0.661** | **<0.001** | **0.730** | **<0.001** |
|  | Stage | **0.019** | **0.006** | 0.012 | 0.649 |
| Cohort 2 | Subject | **0.683** | **<0.001** | **0.741** | **<0.001** |
|  | Stage | **0.013** | **0.010** | 0.017 | 0.289 |

Bold numbers are below P < 0.05. adonis model 2 (data ~ Subject + Stage, permutations = 999, method="bray", by="margin")

**J.** **Intragroup differences between stages of the diet using permutational pairwise testing of beta dispersions from Bray Curtis dissimilarities**

| **Cohort** | **Source** | **Intragroup** | **After-Before** | **After-During** | **Before-During** |
| --- | --- | --- | --- | --- | --- |
| One | Fecal | White | 0.249 | 0.306 | 0.306 |
| One | Fecal | Black | 0.938 | 0.938 | 0.938 |
| Two | Fecal | White | 0.888 | 0.888 | 0.888 |
| Two | Fecal | Black | 0.931 | 0.931 | 0.931 |
| One | Oral | White | 0.728 |  |  |
| One | Oral | Black | 0.924 |  |  |
| Two | Oral | White | 0.213 |  |  |
| Two | Oral | Black | 0.957 |  |  |

FDR-adjusted P-values shown following permutational pairwise testing of multivariate beta dispersions of Bray Curtis dissimilarities. Performed with vegan::permutest(pairwise = T, betadisper(vegdist(data, method='bray')))

**K.** **Intergroup differences between stages of the diet using permutational pairwise testing of beta dispersions from Bray Curtis dissimilarities.**

| **Cohort** | **Source** | **Intergroup** | **After-Before** | **After-During** | **Before-During** |
| --- | --- | --- | --- | --- | --- |
| One | Fecal | Between Ethnicities | 0.784 | 0.784 | 0.784 |
| Two | Fecal | Between Ethnicities | 0.923 | 0.923 | 0.923 |
| One | Oral | Between Ethnicities | 0.873 |  |  |
| Two | Oral | Between Ethnicities | 0.284 |  |  |

FDR-adjusted P-values shown following permutational pairwise testing of multivariate beta dispersions of Bray Curtis dissimilarities. Performed with vegan::permutest(pairwise = T, betadisper(vegdist(data, method='bray')))
