# Supplementary material for: Individuality and ethnicity eclipse a short-term dietary intervention in shaping microbiomes and viromes
Source: PLoS Biol. 2022 Aug 23;20(8):e3001758. doi: 10.1371/journal.pbio.3001758 (PMC9397868; doi:10.1371/journal.pbio.3001758)
Supplement: S3 Table — (DOCX) [file pbio.3001758.s017.docx]

**S3 Table.** **Factors associated with gut and oral microbiomes in both cohorts from assembly-free analysis**

|  | Gut microbiome | | | | Oral microbiome | | | |
| --- | --- | --- | --- | --- | --- | --- | --- | --- |
|  | R^2^ | P | R^2^ | P | R^2^ | P | R^2^ | P |
| **Taxonomy** | Bray-Curtis | | Binary Jaccard | | Bray-Curtis | | Binary Jaccard | |
| Ethnicity | **0.033** | **<0.001** | **0.039** | **<0.001** | **0.055** | **<0.001** | **0.036** | **0.002** |
| Antibiotic use | **0.012** | **0.004** | **0.024** | **<0.001** | **0.044** | **0.002** | **0.026** | **0.021** |
| Hormonal contraceptive | **0.030** | **<0.001** | **0.029** | **<0.001** | 0.011 | 0.538 | **0.025** | **0.018** |
| Cohort | **0.037** | **<0.001** | **0.037** | **<0.001** | **0.023** | **0.048** | **0.021** | **0.017** |
| **COGs** | Bray-Curtis | | Binary Jaccard | | Bray-Curtis | | Binary Jaccard | |
| Ethnicity | **0.019** | **<0.001** | **0.030** | **<0.001** | **0.035** | **0.019** | 0.019 | 0.098 |
| Antibiotic use | **0.016** | **0.002** | **0.029** | **<0.001** | **0.056** | **0.012** | **0.055** | **0.004** |
| Hormonal contraceptive | **0.022** | **<0.001** | **0.025** | **<0.001** | 0.013 | 0.405 | **0.031** | **0.008** |
| Cohort | **0.048** | **<0.001** | **0.062** | **<0.001** | 0.028 | 0.061 | **0.057** | **<0.001** |
| **ARGs** | Bray-Curtis | | Binary Jaccard | | Bray-Curtis | | Binary Jaccard | |
| Ethnicity | **0.022** | **<0.001** | **0.036** | **<0.001** | **0.031** | **0.021** | **0.040** | **<0.001** |
| Antibiotic use | **0.016** | **<0.001** | **0.027** | **<0.001** | **0.043** | **0.005** | 0.020 | 0.078 |
| Hormonal contraceptive | **0.019** | **<0.001** | **0.015** | **0.002** | 0.021 | 0.118 | **0.022** | **0.047** |
| Cohort | **0.042** | **<0.001** | **0.030** | **<0.001** | **0.034** | **0.006** | **0.019** | **0.047** |
| **KEGG** | Bray-Curtis | | Binary Jaccard | | Bray-Curtis | | Binary Jaccard | |
| Ethnicity | **0.019** | **0.006** | **0.051** | **<0.001** | **0.037** | **0.011** | 0.022 | 0.087 |
| Antibiotic use | 0.010 | 0.061 | **0.036** | **<0.001** | **0.036** | **0.015** | **0.029** | **0.034** |
| Hormonal contraceptive | **0.021** | **0.003** | **0.016** | **<0.001** | 0.013 | 0.459 | 0.023 | 0.083 |
| Cohort | **0.027** | **<0.001** | **0.032** | **<0.001** | **0.038** | **0.012** | **0.032** | **0.011** |

Multivariable permutational analysis of variance (PERMANOVA) using Bray-Curtis distance and binary Jaccard distance matrices. Bray-Curtis distance: adonis2(data ~ Ethnicity + Antibiotic Use + Hormonal Contraceptive + Cohort, permutations=perm, method="bray", by="margin"); binary Jaccard distance: adonis2(data ~ Ethnicity + Antibiotic Use + Hormonal Contraceptive + Cohort, permutations=perm, method="jaccards", binary=TRUE, by="margin"); where perm = with(data, how(nperm = 999, blocks=Day)). Bold numbers are below P < 0.05.
