# Supplementary material for: Individuality and ethnicity eclipse a short-term dietary intervention in shaping microbiomes and viromes
Source: PLoS Biol. 2022 Aug 23;20(8):e3001758. doi: 10.1371/journal.pbio.3001758 (PMC9397868; doi:10.1371/journal.pbio.3001758)
Supplement: S4 Table — (A) Cohort 1 from assembly-based analysis; (B) cohort 2 from assembly-based analysis; (C) cohort 1 from assembly-free analysis; (D) cohort 2 from assembly-free analysis; (E) both cohorts from assembly-free analysis. (DOCX) [file pbio.3001758.s018.docx]

**S4 Table. Multivariate homogeneity of groups dispersion results for gut and oral microbiomes gut viromes.** A. Cohort 1 from assembly-based analysis; B. Cohort 2 from assembly-based analysis; C. Cohort 1 from assembly-free analysis; D. Cohort 2 from assembly-free analysis; E. Both cohorts from assembly-free analysis.

**A.** **Multivariate homogeneity of groups dispersion results for gut and oral microbiomes gut viromes in cohort 1 from assembly-based analysis (p-value).**

|  | Gut microbiome | | Oral microbiome | | Gut virome | |
| --- | --- | --- | --- | --- | --- | --- |
|  | Bray-Curtis | Binary Jaccard | Bray-Curtis | Binary Jaccard | Bray-Curtis | Binary Jaccard |
| **Taxonomy** |  |  |  |  |  |  |
| Ethnicity | **<0.001** | 0.788 | 0.296 | 0.571 | **0.020** | **0.029** |
| Stage | 0.844 | 0.618 | 0.954 | 0.659 | 0.777 | 0.957 |
| Antibiotic use^*^ | **<0.001** | **0.006** | 0.635 | 0.080 | **0.019** | **0.011** |
| Hormonal contraceptive^**^ | **0.021** | **<0.001** | 0.167 | 0.340 | **0.019** | 0.879 |
| Subject | **0.002** | **<0.001** | **<0.001** | **<0.001** | **<0.001** | **<0.001** |
| **COGs** |  |  |  |  |  |  |
| Ethnicity | **<0.001** | NA | 0.101 | NA | **0.001** | **<0.001** |
| Stage | 0.612 | NA | 0.951 | NA | 0.689 | 0.854 |
| Antibiotic use^*^ | **0.002** | NA | 0.198 | NA | **0.003** | 0.135 |
| Hormonal contraceptive^**^ | **<0.001** | NA | 0.057 | NA | 0.759 | 0.109 |
| Subject | 0.105 | NA | **<0.001** | NA | **0.010** | **<0.001** |
| **ARGs** |  |  |  |  |  |  |
| Ethnicity | **<0.001** | **0.008** | 0.085 | 0.384 | 0.790 | **0.008** |
| Stage | 0.800 | 0.378 | 0.973 | 0.748 | 0.650 | 0.622 |
| Antibiotic use^*^ | **0.004** | 0.985 | 0.205 | 0.662 | 0.452 | 0.685 |
| Hormonal contraceptive^**^ | **<0.001** | 0.250 | 0.062 | 0.670 | 0.080 | 0180 |
| Subject | 0.155 | 0.100 | **<0.001** | **<0.001** | **0.002** | **0.004** |
| **CAZymes** |  |  |  |  |  |  |
| Ethnicity | **<0.001** | 0.289 | **0.018** | 0.349 | 0.217 | **0.015** |
| Stage | 0.662 | 0.603 | 0.778 | 0.426 | 0.643 | 0.987 |
| Antibiotic use^*^ | **0.050** | 0.289 | 0.252 | 0.833 | 0.239 | 0.402 |
| Hormonal contraceptive^**^ | 0.057 | 0.381 | 0.126 | 0.704 | 0.096 | 0.060 |
| Subject | **0.009** | 0.318 | **<0.001** | **<0.001** | **<0.001** | **<0.001** |
| **KEGG** |  |  |  |  |  |  |
| Ethnicity | **0.007** | **<0.001** |  |  |  |  |
| Stage | 0.645 | 0.963 |  |  |  |  |
| Antibiotic use^*^ | **0.002** | **0.005** |  |  |  |  |
| Hormonal contraceptive^**^ | 0.385 | **0.002** |  |  |  |  |
| Subject | **0.005** | **<0.001** |  |  |  |  |

Multivariate homogeneity of groups dispersions using Bray-Curtis distance and Binary Jaccard distance matrices (Betadisper; Factors tested independently: Ethnicity, Stage, Antibiotic use, Hormonal contraceptive, Subject). ^*^the use of antibiotics within the past year: 9(yes):10(no); ^**^the use of hormonal contraceptive: 8(yes):11(no) (see Table S1). NA denotes all functional categories of COGs are present in gut and oral microbiomes. CAZymes in gut viromes indicate peptidoglycanase. Bold numbers are below P < 0.05.

**B.** **Multivariate homogeneity of groups dispersion results for gut and oral microbiomes and gut viromes in cohort 2 from assembly-based analysis (p-value).**

|  | Gut microbiome | | Oral microbiome | | Gut virome | |
| --- | --- | --- | --- | --- | --- | --- |
|  | Bray-Curtis | Binary Jaccard | Bray-Curtis | Binary Jaccard | Bray-Curtis | Binary Jaccard |
| **Taxonomy** |  |  |  |  |  |  |
| Ethnicity | 0.114 | 0.468 | 0.107 | 0.569 | 0.140 | 0.240 |
| Stage | 0.968 | 0.635 | 0.385 | 0.971 | 0.930 | 0.788 |
| Antibiotic use^*^ | 0.209 | 0.880 | 0.169 | 0.630 | 0.753 | 0.385 |
| Hormonal contraceptive^**^ | 0.699 | 0.192 | 0.156 | 0.113 | 0.251 | 0.436 |
| Subject | 0.613 | **<0.001** | **<0.001** | **<0.001** | 0.186 | 0.132 |
| **COGs** |  |  |  |  |  |  |
| Ethnicity | **0.018** | NA | **0.002** | NA | 0.197 | 0.201 |
| Stage | 0.886 | NA | 0.852 | NA | 0.408 | 0.913 |
| Antibiotic use^*^ | 0.327 | NA | 0.108 | NA | 0.886 | 0.683 |
| Hormonal contraceptive^**^ | 0.405 | NA | **0.004** | NA | 0.959 | 0.999 |
| Subject | **0.010** | NA | **<0.001** | NA | **0.032** | **0.014** |
| **ARGs** |  |  |  |  |  |  |
| Ethnicity | 0.650 | NA | **0.015** | 0.641 | 0.071 | 0.394 |
| Stage | 0.646 | NA | 0.935 | 0.384 | 0.684 | 0.951 |
| Antibiotic use^*^ | 0.897 | NA | 0.185 | 0.915 | 0.824 | 0.172 |
| Hormonal contraceptive^**^ | 0.760 | NA | **0.007** | 0.641 | 0.477 | 0.375 |
| Subject | **0.022** | NA | **<0.001** | **<0.001** | **0.026** | 0.245 |
| **CAZymes** |  |  |  |  |  |  |
| Ethnicity | 0.195 | 0.208 | 0.530 | 0.167 | **0.017** | 0.640 |
| Stage | 0.964 | 0.393 | 0.359 | 1 | 0.456 | 0.446 |
| Antibiotic use^*^ | 0.263 | **0.044** | 0.556 | 0.348 | 0.750 | 0.913 |
| Hormonal contraceptive^**^ | 0.347 | 0.096 | **0.019** | 0.262 | **<0.001** | 0.176 |
| Subject | 0.937 | 0.245 | **<0.001** | **<0.001** | 0.218 | 0.482 |
| **KEGG** |  |  |  |  |  |  |
| Ethnicity | 0.127 | 0.789 |  |  |  |  |
| Stage | 0.995 | 0.423 |  |  |  |  |
| Antibiotic use^*^ | 0.971 | **0.015** |  |  |  |  |
| Hormonal contraceptive^**^ | 0.901 | 0.805 |  |  |  |  |
| Subject | 0.065 | **<0.001** |  |  |  |  |

Multivariate homogeneity of groups dispersions using Bray-Curtis distance and Binary Jaccard distance matrices (Betadisper; Factors tested independently: Ethnicity, Stage, Antibiotic use, Hormonal contraceptive, Subject). ^*^the use of antibiotics within the past year: 10(yes):7(no); ^**^the use of hormonal contraceptive: 6(yes):11(no) (see Table S1). NA denotes all functional categories of COGs are present in gut and oral microbiomes. CAZymes in gut viromes indicate peptidoglycanase. Bold numbers are below P < 0.05.

**C.** **Multivariate homogeneity of groups dispersion results for gut and oral microbiomes in cohort 1 from assembly-free analysis (p-value).**

|  | Gut microbiome | | Oral microbiome | |
| --- | --- | --- | --- | --- |
|  | Bray-Curtis | Binary Jaccard | Bray-Curtis | Binary Jaccard |
| **Taxonomy** |  |  |  |  |
| Ethnicity | **<0.001** | 0.204 | 0.724 | 0.079 |
| Stage | 0.836 | 0.686 | 0.873 | 0.811 |
| Antibiotic use^*^ | **<0.001** | **<0.001** | 0.791 | 0.120 |
| Hormonal contraceptive^**^ | **0.018** | **<0.001** | 0.173 | 0.896 |
| Subject | **0.003** | **0.023** | **<0.001** | **<0.001** |
| **COGs** |  |  |  |  |
| Ethnicity | **<0.001** | 0.172 | 0.658 | **0.040** |
| Stage | 0.934 | 0.777 | 0.724 | 0.894 |
| Antibiotic use^*^ | **<0.001** | **0.011** | 0.804 | 0.103 |
| Hormonal contraceptive^**^ | **0.004** | **<0.001** | 0.058 | 0.979 |
| Subject | **0.003** | **<0.001** | **<0.001** | **<0.001** |
| **ARGs** |  |  |  |  |
| Ethnicity | **0.045** | **<0.001** | 0.109 | 0.070 |
| Stage | 0.790 | 0.425 | 0.617 | 0.260 |
| Antibiotic use^*^ | 0.144 | 0.369 | 0.339 | 0.673 |
| Hormonal contraceptive^**^ | 0.578 | 0.293 | 0.341 | 0.215 |
| Subject | **<0.001** | **<0.001** | **<0.001** | **<0.001** |
| **KEGG** |  |  |  |  |
| Ethnicity | **<0.001** | **<0.001** | 0.473 | 0.085 |
| Stage | 0.876 | 0.499 | 0.801 | 0.994 |
| Antibiotic use^*^ | **<0.001** | 0.763 | 0.806 | 0.562 |
| Hormonal contraceptive^**^ | **<0.001** | 0.665 | 0.159 | 0.278 |
| Subject | **<0.001** | **<0.001** | **<0.001** | **<0.001** |

Multivariate homogeneity of groups dispersions using Bray-Curtis distance and Binary Jaccard distance matrices (Betadisper; Factors tested independently: Ethnicity, Stage, Antibiotic use, Hormonal contraceptive, Subject). ^*^the use of antibiotics within the past year: 10(yes):7(no); ^**^the use of hormonal contraceptive: 6(yes):11(no) (see Table S1). NA denotes all functional categories of COGs are present in gut and oral microbiomes. Bold numbers are below P < 0.05.

**D.** **Multivariate homogeneity of groups dispersion results for gut and oral microbiomes in cohort 2 from assembly-free analysis (p-value).**

|  | Gut microbiome | | Oral microbiome | |
| --- | --- | --- | --- | --- |
|  | Bray-Curtis | Binary Jaccard | Bray-Curtis | Binary Jaccard |
| **Taxonomy** |  |  |  |  |
| Ethnicity | 0.134 | 0.547 | 0.192 | 0.321 |
| Stage | 0.872 | 0.500 | 0.284 | 0.668 |
| Antibiotic use^*^ | 0.108 | 0.239 | 0.160 | 0.624 |
| Hormonal contraceptive^**^ | 0.123 | **0.041** | 0.083 | **0.018** |
| Subject | 0.836 | 0.171 | **<0.001** | **<0.001** |
| **COGs** |  |  |  |  |
| Ethnicity | **0.025** | **0.006** | 0.078 | 0.056 |
| Stage | 0.658 | 0.833 | 0.337 | 0.340 |
| Antibiotic use^*^ | 0.356 | **0.007** | 0.338 | 0.328 |
| Hormonal contraceptive^**^ | 0.654 | **<0.001** | 0.169 | **0.013** |
| Subject | 0.085 | **0.005** | **<0.001** | **<0.001** |
| **ARGs** |  |  |  |  |
| Ethnicity | 0.387 | 0.509 | 0.656 | 0.453 |
| Stage | 0.481 | **0.009** | 0.215 | 0.839 |
| Antibiotic use^*^ | **0.042** | **<0.001** | 0.296 | 0.109 |
| Hormonal contraceptive^**^ | **0.004** | **0.040** | 0.337 | 0.716 |
| Subject | 0.620 | **0.050** | **<0.001** | **<0.001** |
| **KEGG** |  |  |  |  |
| Ethnicity | 0.065 | 0.659 | 0.235 | 0.059 |
| Stage | 0.598 | 0.414 | 0.575 | 0.540 |
| Antibiotic use^*^ | 0.626 | **<0.001** | 0.099 | 0.349 |
| Hormonal contraceptive^**^ | 0.697 | **<0.001** | 0.371 | **0.046** |
| Subject | **0.018** | **<0.001** | **<0.001** | **<0.001** |

Multivariate homogeneity of groups dispersions using Bray-Curtis distance and Binary Jaccard distance matrices (Betadisper; Factors tested independently: Ethnicity, Stage, Antibiotic use, Hormonal contraceptive, Subject). ^*^the use of antibiotics within the past year: 10(yes):7(no); ^**^the use of hormonal contraceptive: 6(yes):11(no) (see Table S1). NA denotes all functional categories of COGs are present in gut and oral microbiomes. Bold numbers are below P < 0.05.

**E.** **Multivariate homogeneity of groups dispersion results for gut and oral microbiomes in both cohorts from assembly-free analysis (p-value).**

|  | Gut microbiome | | Oral microbiome | |
| --- | --- | --- | --- | --- |
|  | Bray-Curtis | Binary Jaccard | Bray-Curtis | Binary Jaccard |
| **Taxonomy** |  |  |  |  |
| Ethnicity | **0.007** | 0.104 | 0.630 | 0.075 |
| Stage | 0.773 | 0.532 | 0.496 | 0.635 |
| Antibiotic use | 0.398 | 0.725 | 0.330 | 0.087 |
| Hormonal contraceptive | **0.002** | **<0.001** | 0.056 | 0.242 |
| Cohort | 0.842 | **<0.001** | 0.113 | **0.003** |
| Subject | **0.004** | **<0.001** | **<0.001** | **<0.001** |
| **COGs** |  |  |  |  |
| Ethnicity | 0.099 | 0.556 | 0.087 | **0.018** |
| Stage | 0.820 | 0.331 | 0.674 | 0.394 |
| Antibiotic use | 0.213 | 0.151 | 0.091 | 0.134 |
| Hormonal contraceptive | **0.014** | **<0.001** | 0.055 | 0.105 |
| Cohort | **<0.001** | **<0.001** | **<0.001** | **0.009** |
| Subject | **<0.001** | **<0.001** | **<0.001** | **<0.001** |
| **ARGs** |  |  |  |  |
| Ethnicity | 0.707 | **0.002** | 0.121 | 0.441 |
| Stage | 0.906 | 0.054 | 0.192 | 0.254 |
| Antibiotic use | 0.558 | **<0.001** | 0.885 | **0.008** |
| Hormonal contraceptive | 0.272 | 0.084 | 0.223 | 0.130 |
| Cohort | 0.385 | **<0.001** | 0.179 | 0.635 |
| Subject | **<0.001** | **<0.001** | **<0.001** | **<0.001** |
| **KEGG** |  |  |  |  |
| Ethnicity | **0.039** | **<0.001** | 0.906 | **0.025** |
| Stage | 0.943 | **<0.001** | 0.241 | **0.046** |
| Antibiotic use | **0.010** | **0.001** | 0.858 | 0.795 |
| Hormonal contraceptive | **0.014** | 0.195 | 0.156 | 0.149 |
| Cohort | 0.771 | **<0.001** | **0.045** | **0.006** |
| Subject | **<0.001** | **<0.001** | **<0.001** | **<0.001** |

Multivariate homogeneity of groups dispersions using Bray-Curtis distance and Binary Jaccard distance matrices (Betadisper; Factors tested independently: Ethnicity, Stage, Antibiotic use, Hormonal contraceptive, Subject). Bold numbers are below P < 0.05.
