# Supplementary material for: Individuality and ethnicity eclipse a short-term dietary intervention in shaping microbiomes and viromes
Source: PLoS Biol. 2022 Aug 23;20(8):e3001758. doi: 10.1371/journal.pbio.3001758 (PMC9397868; doi:10.1371/journal.pbio.3001758)
Supplement: S5 Table — Alpha diversity (Shannon diversity index) is stable in response to diet intervention in both cohorts based on (A) assembly-based analysis and (B) assembly-free data. (DOCX) [file pbio.3001758.s019.docx]

**S5 Table. Alpha diversity (Shannon diversity index) is stable in response to diet intervention in both cohorts.**

**A. Alpha diversity (Shannon diversity index) is stable in response to diet intervention in both cohorts based on assembly-based analysis.**

|  | Gut microbiome | | Oral microbiome | |
| --- | --- | --- | --- | --- |
|  | W/ χ^2^ | *P* | W/ χ^2^ | *P* |
| **Cohort 1** |  |  |  |  |
| Ethnicity | 1564 | 0.227 | 83 | 0.008 |
| Stage | 1.010 | 0.604 | 188 | 0.616 |
| **Cohort 2** |  |  |  |  |
| Ethnicity | 1379 | 0.967 | 82 | 0.044 |
| Stage | 1.448 | 0.485 | 144 | 0.999 |

Tests for ethnicity were performed with a Wilcoxon rank sum test and tests for stage were performed with a Kruskal-Wallis rank sum test for gut microbiome data and a Wilcoxon rank sum test for oral microbiome data.

**B. Alpha diversity (Shannon diversity index) is stable in response to diet intervention in both cohorts based on assembly-free data.**

|  | Gut microbiome | | Oral microbiome | |
| --- | --- | --- | --- | --- |
|  | W/ χ^2^ | *P* | W/ χ^2^ | *P* |
| **Cohort 1** |  |  |  |  |
| Ethnicity | 1671 | 0.087 | 200 | 0.569 |
| Stage | 2.268 | 0.322 | 174 | 0.861 |
| **Cohort 2** |  |  |  |  |
| Ethnicity | 1578 | 0.225 | 144 | 0.903 |
| Stage | 1.049 | 0.592 | 144 | 0.999 |

Tests for ethnicity were performed with a Wilcoxon rank sum test and tests for stage were performed with a Kruskal-Wallis rank sum test for gut microbiome data and a Wilcoxon rank sum test for oral microbiome data.
