# Supplementary material for: Individuality and ethnicity eclipse a short-term dietary intervention in shaping microbiomes and viromes
Source: PLoS Biol. 2022 Aug 23;20(8):e3001758. doi: 10.1371/journal.pbio.3001758 (PMC9397868; doi:10.1371/journal.pbio.3001758)
Supplement: S6 Table — (DOCX) [file pbio.3001758.s020.docx]

**S6 Table.** **Contrasting ethnic difference of the heritable taxa in oral and gut microbiome among two ethnicities across the dietary intervention** (assembly-based analysis, FDR <0.05, LinDA)

| Taxa | Cohort 1 | | | |  | Cohort 2 | | | |
| --- | --- | --- | --- | --- | --- | --- | --- | --- | --- |
|  | Fecal microbiome | | Oral microbiome | |  | Fecal microbiome | | Oral microbiome | |
|  | p.value | p.fdr | p.value | p.fdr |  | p.value | p.fdr | p.value | p.fdr |
| *Veillonella*_G | 0.403 | 0.726 | 0.005 | 0.046 |  | 0.219 | 0.394 | 0.058 | 0.522 |
| Christensenellaceae_F | 0.134 | 0.369 | 0.428 | 0.642 |  | 0.009 | 0.038 | 0.206 | 0.587 |
| Coriobacteriaceae_F | 0.137 | 0.369 | 0.814 | 0.923 |  | 0.854 | 0.961 | 0.487 | 0.641 |
| Odoribacteraceae_F | 0.058 | 0.369 | 0.053 | 0.159 |  | 0.067 | 0.193 | 0.261 | 0.587 |
| Peptococcaceae_F | 0.880 | 0.880 | 0.821 | 0.923 |  | 0.796 | 0.961 | 0.498 | 0.641 |
| Rikenellaceae_F | 0.614 | 0.864 | 0.159 | 0.358 |  | 0.00002 | 0.0002 | 0.820 | 0.844 |
| Verrucomicrobiaceae_F | 0.768 | 0.864 | 0.288 | 0.518 |  | 0.967 | 0.967 | 0.844 | 0.844 |
| Victivallaceae_F | 0.767 | 0.864 | 0.017 | 0.078 |  | 0.086 | 0.193 | 0.160 | 0.587 |
| Clostridiales_O | 0.164 | 0.369 | 0.976 | 0.976 |  | 0.268 | 0.403 | 0.397 | 0.641 |

Significance test (LinDA function in MicrobiomeStat package fitting mixed-effect models '~ Ethnicity + Antibiotic Use + Hormonal Contraceptive + (1|Day)') for centered log-ratio transformed abundance between two ethnicities._G denotes Genus, _F denotes Family, _O denotes Order. Pink, more abundant in Black participants; blue, more abundant in White participants.
