# Supplementary material for: Individuality and ethnicity eclipse a short-term dietary intervention in shaping microbiomes and viromes
Source: PLoS Biol. 2022 Aug 23;20(8):e3001758. doi: 10.1371/journal.pbio.3001758 (PMC9397868; doi:10.1371/journal.pbio.3001758)
Supplement: S7 Table — Urine (A) and plasma (B) super pathway analysis by ethnicity. (DOCX) [file pbio.3001758.s021.docx]

**S7 Table**. **Urine (A) and Plasma (B) Super Pathway Analysis by Ethnicity**

**A.** Urine Super Pathway Analysis by Ethnicity

| **Cohort1** | **P-Value (Before Diet)** | **P-Value (After Diet)** |
| --- | --- | --- |
| Amino Acid | NS | NS |
| Cofactors and Vitamins | NS | NS |
| Energy | NS | NS |
| Lipid | NS | NS |
| Nucleotide | NS | NS |
| Partially Characterized Molecules | NS | NS |
| Carbohydrate | NS | NS |
| Peptide | NS | NS |
| Xenobiotics | NS | NS |
| **Cohort2** |  |  |
| Amino Acid | 0.01666667 | NS |
| Cofactors and Vitamins | NS | NS |
| Energy | NS | NS |
| Lipid | NS | NS |
| Nucleotide | NS | NS |
| Partially Characterized Molecules | 0.00555556 | 0.00555556 |
| Carbohydrate | NS | NS |
| Peptide | 0.01666667 | 0.01666667 |
| Xenobiotics | NS | NS |

**B.** Plasma Super Pathway Analysis by Ethnicity

| **Cohort 1** | **P-Value (Before Diet)** | **P-Value (After Diet)** |
| --- | --- | --- |
| Amino Acid | NS | NS |
| Cofactors and Vitamins | NS | NS |
| Energy | NS | NS |
| Lipid | NS | NS |
| Nucleotide | NS | NS |
| Partially Characterized Molecules | NS | NS |
| Carbohydrate | NS | NS |
| Peptide | NS | NS |
| Xenobiotics | NS | NS |
| **Cohort 2** |  |  |
| Amino Acid | 0.01666667 | 0.00555556 |
| Cofactors and Vitamins | NS | NS |
| Energy | NS | NS |
| Lipid | NS | NS |
| Nucleotide | NS | NS |
| Partially Characterized Molecules | 0.00555556 | NS |
| Carbohydrate | NS | NS |
| Peptide | NS | NS |
| Xenobiotics | NS | NS |
