# Supplementary material for: Individuality and ethnicity eclipse a short-term dietary intervention in shaping microbiomes and viromes
Source: PLoS Biol. 2022 Aug 23;20(8):e3001758. doi: 10.1371/journal.pbio.3001758 (PMC9397868; doi:10.1371/journal.pbio.3001758)
Supplement: S8 Table — (A) Cohort 1 urine; (B) cohort 2 urine; (C) cohort 1 plasma; (D) cohort 2 plasma. (DOCX) [file pbio.3001758.s022.docx]

**S8 Table. Metabolome pathway analysis.** A Cohort 1 Urine; B. Cohort 2 Urine; C. Cohort 1 Plasma; D. Cohort 2 Plasma.

**A** Urine metabolome pathway analysis- Cohort 1

| **Pathway Name** | **Holm P** | **FDR** |
| --- | --- | --- |
| Caffeine metabolism | 2.17E-05 | 2.17E-05 |
| Histidine metabolism | 0.18855 | 0.095411 |
| beta-Alanine metabolism | 1 | 1 |
| Nicotinate and nicotinamide metabolism | 1 | 1 |
| Pantothenate and CoA biosynthesis | 1 | 1 |
| Fructose and mannose metabolism | 1 | 1 |
| Citrate cycle (TCA cycle) | 1 | 1 |
| Galactose metabolism | 1 | 1 |
| Alanine, aspartate and glutamate metabolism | 1 | 1 |
| Glyoxylate and dicarboxylate metabolism | 1 | 1 |
| Glycine, serine and threonine metabolism | 1 | 1 |
| Amino sugar and nucleotide sugar metabolism | 1 | 1 |
| Pyrimidine metabolism | 1 | 1 |
| Fatty acid biosynthesis | 1 | 1 |

**B** Urine metabolome pathway analysis- Cohort 2

| **Pathway Name** | **Holm P** | **FDR** |
| --- | --- | --- |
| Citrate cycle (TCA cycle) | 0.010697 | 0.010697 |
| Alanine, aspartate and glutamate metabolism | 0.041437 | 0.020968 |
| Arginine biosynthesis | 1 | 0.34385 |
| Butanoate metabolism | 1 | 0.34385 |
| Histidine metabolism | 1 | 0.34385 |
| D-Arginine and D-ornithine metabolism | 1 | 0.77881 |
| D-Glutamine and D-glutamate metabolism | 1 | 0.98789 |
| Valine, leucine and isoleucine biosynthesis | 1 | 1 |
| Tyrosine metabolism | 1 | 1 |
| Caffeine metabolism | 1 | 1 |
| Phenylalanine metabolism | 1 | 1 |
| Pantothenate and CoA biosynthesis | 1 | 1 |
| beta-Alanine metabolism | 1 | 1 |
| Pyruvate metabolism | 1 | 1 |
| Propanoate metabolism | 1 | 1 |
| Glyoxylate and dicarboxylate metabolism | 1 | 1 |
| Cysteine and methionine metabolism | 1 | 1 |
| Valine, leucine and isoleucine degradation | 1 | 1 |
| Tryptophan metabolism | 1 | 1 |

**C** Plasma metabolome pathway analysis- Cohort 1

| **Pathway Name** | **Holm P** | **FDR** |
| --- | --- | --- |
| Caffeine metabolism | 4.69E-09 | 4.69E-09 |
| Lysine degradation | 1 | 1 |
| Glycine, serine and threonine metabolism | 1 | 1 |
| Valine, leucine and isoleucine biosynthesis | 1 | 1 |
| Biotin metabolism | 1 | 1 |
| Aminoacyl-tRNA biosynthesis | 1 | 1 |
| Histidine metabolism | 1 | 1 |
| Ether lipid metabolism | 1 | 1 |
| Porphyrin and chlorophyll metabolism | 1 | 1 |
| Cysteine and methionine metabolism | 1 | 1 |
| Glycerophospholipid metabolism | 1 | 1 |
| Arginine and proline metabolism | 1 | 1 |
| Pyrimidine metabolism | 1 | 1 |
| Tryptophan metabolism | 1 | 1 |

**D** Plasma metabolome pathway analysis- Cohort 2

| **Pathway Name** | **Holm P** | **FDR** |
| --- | --- | --- |
| Caffeine metabolism | 0.0016174 | 0.0016174 |
| Lysine degradation | 0.079194 | 0.040074 |
| Aminoacyl-tRNA biosynthesis | 1 | 1 |
| Valine, leucine and isoleucine biosynthesis | 1 | 1 |
| Ascorbate and aldarate metabolism | 1 | 1 |
| Biotin metabolism | 1 | 1 |
| Arginine biosynthesis | 1 | 1 |
| Histidine metabolism | 1 | 1 |
| Pentose and glucuronate interconversions | 1 | 1 |
| Pantothenate and CoA biosynthesis | 1 | 1 |
| Selenocompound metabolism | 1 | 1 |
| Alanine, aspartate and glutamate metabolism | 1 | 1 |
| Inositol phosphate metabolism | 1 | 1 |
| Steroid hormone biosynthesis | 1 | 1 |
| Arginine and proline metabolism | 1 | 1 |
| Valine, leucine and isoleucine degradation | 1 | 1 |
| Tryptophan metabolism | 1 | 1 |
